# Photographic documentation of melanism in bobcats (*Lynx rufus*) in the Greater Everglades

**DOI:** 10.1002/ece3.10754

**Published:** 2024-01-16

**Authors:** Aidan B. Branney, Heather N. Abernathy, L. Mike Conner, Elina Garrison, Michael J. Cherry

**Affiliations:** ^1^ Caesar Kleberg Wildlife Research Institute Texas A&M University‐Kingsville Kingsville Texas USA; ^2^ Haub School of Environment and Natural Resources University of Wyoming Laramie Wyoming USA; ^3^ The Jones Center at Ichauway Newton Georgia USA; ^4^ Florida Fish and Wildlife Conservation Commission Gainesville Florida USA; ^5^ Present address: California Department of Fish and Wildlife Rancho Cardova California USA

**Keywords:** bobcat, fire regime, Florida panther, Gloger's rule, Greater Everglades, melanism

## Abstract

We document the presence of bobcats (*Lynx rufus*) that demonstrate melanism in the Greater Everglades. The South Florida landscape is driven by a myriad of disturbance regimes particularly that of short fire intervals. We monitored 180 camera traps for 3 years and obtained 9503 photographs of bobcats 25 (<0.5%) of these detections included melanistic individuals. Our observations and historical accounts suggest melanism is a phenotype that persists, albeit it at an exceedingly low frequency, in bobcats in the region. While we do not know if the expression of melanism conferred a fitness benefit in our system, the vegetation structure that was characterized by frequently burned uplands and low‐light and densely vegetated swamps produced conditions that may render a benefit from melanism through enhanced crypsis. The investigation of rare phenomenon in ecology is important yet difficult within a given field study, but reporting novel observations, like melanism in bobcats, allows for science to gain insight across studies that would not be otherwise possible.

## Graphical abstract

We report the rare observations of melanistic bobcats in their natural environment within the Greater Everglades Ecosystem. We also discuss the factors that could support melanistic phenotypes within this subtropical environment.

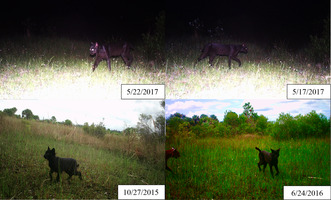

## 1 INTRODUCTION

Ecogeographic rules describe the link between an organism's traits and its environment. This correlation arises from natural selection shaping organismal traits to optimize fitness (Mayr, 1963; VanderWerf, 2012). Variation in overall melanin content has been theorized to be dictated by climate wherein feathers and fur contain greater amounts of melanin and consequently are darker in rainy and warm climates (James, 1991); yet exceptions have been reported and attributed to thermoregulatory advantages (Delhey, 2019; Rising et al., 2009). Moreover, crypsis, which is the ability of the animal to blend in with its surroundings and avoid detection by other animals, may also explain variation in melanin content (da Silva et al., 2017; Guthrie, 1967; Nachman et al., 2003). For example, lava melanism and fire melanism describe an increase in the proportion of populations expressing melanism near lava fields or following fires when dark phenotypes provide an advantage due to enhanced crypsis (Forsman et al., 2011; Guthrie, 1967; Hocking, 1964; Karlsson et al., 2008; Kiltie, 1989). Melanism is frequently observed across mammals and birds (Delhey, 2017; Kingsley et al., 2009; Reissmann et al., 2020; Uy et al., 2016), and in carnivores it is most often documented when the expression of melanism is likely to confer a benefit from increased concealment while stalking prey, a pattern most notable in felids (Eizirik et al., 2003; Schneider et al., 2015).

Currently, 14 of the 41 wild members of Felidae (Kitchener et al., 2017) are known to exhibit melanism, with the greatest instances occurring in jaguars (*Panthera onca*) and leopards (*Panthera pardus*; Graipel et al., 2019). In North America, melanism has been documented in jaguars, jaguarundi (*Puma yagouaroundi*), and bobcats (*Lynx rufus*; McAlpine, 2021). There have been multiple instances of melanistic jaguars and jaguarundis within Central America's low‐light tropical forests (da Silva et al., 2016; Mooring et al., 2020). These findings suggest larger patches of forest, combined with the region's humid climate, likely contribute to the prevalence of felines with darker pelage patterns. This observation aligns with Gloger's rule, which posits that melanin production increases in mammals inhabiting warm and humid climates (Delhey, 2019). The darker pelage patterns provide advantages in terms of both thermoregulation and concealment cover within these environments (Delhey, 2019; Mooring et al., 2020). Thus, the combination of forest patch size and climate may promote darker pelage patterns and the expression of the phenotype could increase fitness for felids within low‐light, tropical systems. However, there are disadvantages to possessing a darker pelage ranging from being a sought out for recreational trapping, loss of ability to communicate, and restrictions to times of day to hunt prey (Graipel et al., 2019; Mooring et al., 2020; Regan & Maer, 1990).

An aspect that has received less emphasis in the literature is the potential contribution of both low‐light forests and fire as drivers of melanism. The Greater Everglades is a semi‐tropical system characterized by a mosaic of thick forested canopy, flooded wetlands, and open upland prairies (Chimney & Goforth, 2001). This ecosystem is sculpted by a myriad of disturbance regimes including hurricanes, wild and prescribed fire, and seasonal flooding (Abernathy et al., 2019, 2022; Lockwood et al., 2003). Importantly, the system is characterized by one of the shortest fire return intervals in North America (Guyette et al., 2012). The fire‐dependent vegetation community requires frequent fires to maintain pyrophilic plant species (Slocum et al., 2003). The presence of seasonal fires contributes to the formation of vegetation mosaics, resulting in increased structural complexity within the region (Ruiz et al., 2013; Smith et al., 2013). This complexity is further influenced by the interactive effects of hydroperiod and fire return interval, which drive variations in plant community composition across the landscape ranging from densely forested communities in dry areas to expansive marshes with varying hydroperiods (Kominoski et al., 2022).

We captured photographic evidence of melanism in bobcats in Florida, USA while conducting a camera trap study. Melanism is a rare phenotype in bobcats and previous reports are primarily from the southeastern edge of the species' distribution. We propose the disturbance regimes in our system created conditions suitable for melanism to provide an adaptive advantage that could explain the persistence of this phenotype within the area.

## 2 MATERIALS AND METHODS

**FIGURE 1:**
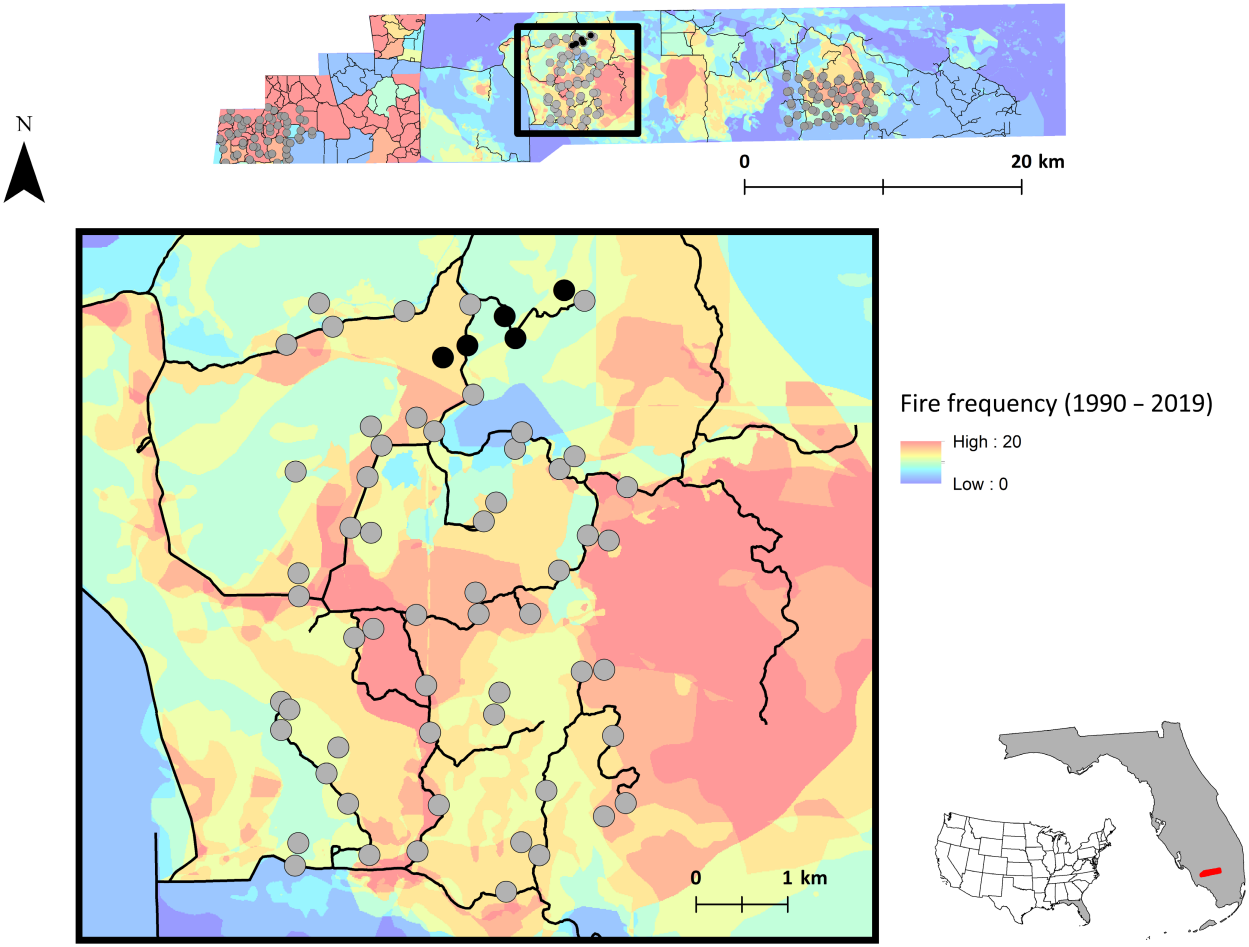
Map of the three camera grids located in the Greater Everglades in South Florida with overlaid heat map of natural and prescribed fire frequency from 1990 to 2019. Cooler colors represent areas of the landscape with relatively fewer fires, while warmer colors represent more fires. Three total camera grids were deployed throughout the study area, on and off trails, where trails are indicated by the black lines on the map. A total of five cameras (black points) out of 60 cameras (gray points) in the central grid detected melanistic individuals during the duration of concurrent research projects from January 2015 to December 2019.

We conducted our field work in the Big Cypress Basin of the Greater Everglades, Florida, USA (Figure 1). The Greater Everglades represents one of the largest patches of continuous wetlands and subtropical forest found in the United States. The region is defined by diverse plant communities including pineland, hardwood hammock, cypress, and mangrove forests as well as coastal and upland prairies. Southwest Florida contains many fire‐dependent system (Main & Richardson, 2002) with fire return intervals ranging from 2 to 10 years (Burch, 2004). Our study area recorded 618 fires over 30 years (1990–2019) with some patches experiencing 20 fires during that time (Figure 1). The mean patch size of wild and prescribed fires was 2.76 km^2^ (range: 0.0051 km^2^, 146.98 km^2^). Fire can have profound effects on the immediate and long‐term space use of wildlife (Cherry et al., 2018; Main & Richardson, 2002).

To study of interactions between white‐tailed deer (*Odocoileus virginianus*) and Florida panther (*Puma concolor coryi*), we deployed 180 motion‐activated white‐flash trail cameras (HCO Outdoor Products, model SG565FV, Norcross, GA, USA) across three distinct study grids. Each camera grid encompassed more than 29 km^2^ separated by at least 13 km. Within each of these grids, we positioned 40 trail cameras along trails (Figure 1) approximately 700 m apart, complemented by an additional 20 camera traps spaced roughly 250 m apart from the nearest on‐trail camera. Cameras were monitored from January 2015 to December 2017, following the maintenance and data retrieval protocols outlined in Crawford et al. (2019).

## 3 RESULTS

We documented wildlife occurrences during 192,089 trap nights between 2015 and 2017. Bobcat detections were relatively evenly distributed throughout the year with no strong seasonality to detection rates. The proportion of cameras with at least one bobcat detected on any given day averaged 5% CI (3.8–5.5). We documented 9503 detections of bobcats, of which 25 were of a bobcat expressing melanism (Appendix S1). We assume the detections were of a single melanistic individual, which we captured first as a juvenile and later as an adult. However, we acknowledge our photographs could be from multiple melanistic bobcats, as the lack of unique spot patterns limited our ability to discern individuals. Of the 60 cameras in the central grid, five cameras detected a melanistic bobcat. The farthest distance between cameras that detected a melanistic bobcat was 1500 m.

**FIGURE 2:**
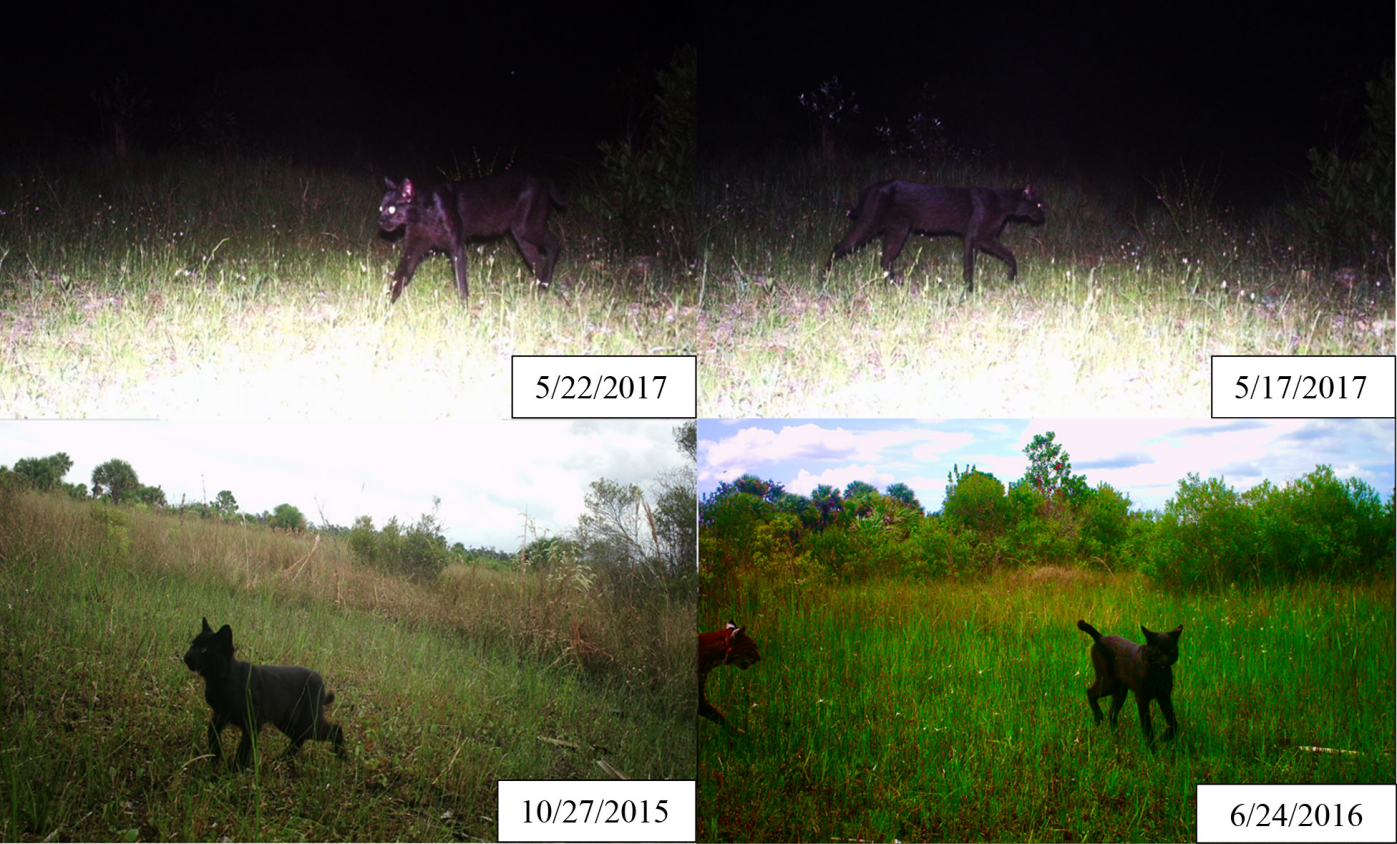
Photographs of four different detections of a melanistic bobcat (*Lynx rufus*) within the Greater Everglades in South Florida collected from January 2015 to December 2017. Photo credits belong to Elina Garrison, FWC.

The photographic evidence, including sequence photos (Figure 2), showcases a melanistic individual navigating its environment. While a few cases of melanistic bobcats have been reported in harvest records and highway mortalities, we believed this to be the first time that the existence of melanistic bobcats has been confirmed through trail camera documentation (Hutchinson & Hutchinson, 2000; McAlpine, 2021; Regan & Maer, 1990; Ulmer, 1941). These observations combined with ecosystem characteristics – frequent fires and low‐light subtropical forests – suggest that melanism may be advantageous for bobcats in this region.

## 4 DISCUSSION

Both fire regimes and forest communities within the Greater Everglades may promote greater melanin content in animals, enhancing crypsis. Melanism has been documented across Florida in other taxa including eastern fox squirrels (*Sciurus niger*; Potash et al., 2020) and coyotes (*Canis latrans*; Caudill & Caudill, 2015). Within the Everglades Region and other fire‐dependent systems, melanism may improve the hunting success of predators and predator evasion by prey in frequently burned patches. However, within our study only 0.2% of our 9503 bobcat detections demonstrated melanism which compared to studies of melanism in jaguars (25%) or melanistic oncilla (32%) (*Leopardus tigrinus*) is extremely low (Graipel et al., 2014; Mooring et al., 2020) and lower than the 8% observed of melanistic coyotes in Florida (Caudill & Caudill, 2015). While most documented observations of melanistic bobcats occur in Florida (Hutchinson & Hutchinson, 2000; Regan & Maer, 1990; Ulmer, 1941), based on our data and historical observations it seems melanism in bobcats is an exceedingly rare phenotype. We were surprised to observe this rare phenomenon in bobcats as it had not been previously reported despite numerous camera trap studies in Florida. We suggest the dense vegetation structure, and black fire scars from frequently low intensity fires within our study system, would offer the added advantage of crypsis while hunting and avoiding larger carnivores.

Research efforts to study the genetic predisposition of melanin content in other neotropical felids have been identified and found the mutations that promote darker pelage patterns across pampas cat (*Leopardus colocola*), kodkod (*Leopardus guigna*), and Geoffroy's cat (*Leopardus geoffroyi*; Schneider et al., 2015). Despite bobcats being one of the widest distributed and most abundant felids in North America (Cerreta et al., 2023; Lavariega et al., 2022; Roberts & Crimmins, 2010), to date the molecular ecology of melanism in bobcats is poorly understood (McAlpine, 2021).

While melanism in bobcats has been reported here and elsewhere (McAlpine, 2021), there is no evidence of melanism in *P. concolor* (Graipel et al., 2019; Pike et al., 1999; Rosatte, 2011; Tischendorf & McAlpine, 1995). The lack of confirmed observations of melanistic mountain lions would also suggest they do not possess the genetic predisposition to exhibit melanism and this is also highly unlikely with Florida panthers (*Puma concolor coryi*) as the population is in a genetic bottleneck (Graipel et al., 2019; Johnson et al., 2010). Given that Florida panthers could benefit from the purported hunting advantages we discuss associated with melanism, the absence of this phenotypic morph in panthers despite widespread folklore is intriguing. Studies of melanism in wild felids is exceedingly difficult due to the low frequency of the expression of the phenotype. This makes observations like ours particularly important. We call for researchers to publish similar observations of melanism so that future studies can compile these observations to identify mechanisms driving the variation in the frequency of occurrence of melanism in populations and the fitness consequences of the expression of the phenotype.

## AUTHOR CONTRIBUTIONS

**Aidan B. Branney:** Conceptualization (lead); data curation (lead); writing – original draft (lead); writing – review and editing (lead). **Heather N. Abernathy:** Formal analysis (equal); methodology (supporting); visualization (equal); writing – review and editing (equal). **L. Mike Conner:** Investigation (equal); writing – review and editing (equal). **Elina Garrison:** Investigation (equal); methodology (equal); project administration (equal); writing – review and editing (equal). **Michael J. Cherry:** Conceptualization (equal); funding acquisition (lead); investigation (equal); project administration (equal); writing – review and editing (equal).

## Supporting information


Appendix S1.

## Data Availability

The data that support the findings of this study are available in the Supporting Information of this article.

## References

[ece310754-bib-0001] Abernathy, H. N. , Chandler, R. B. , Crawford, D. A. , Garrison, E. P. , Conner, L. M. , Miller, K. V. , & Cherry, M. J. (2022). Behavioral responses to ecological disturbances influence predation risk for a capital breeder. Landscape Ecology, 37, 233–248.

[ece310754-bib-0002] Abernathy, H. N. , Crawford, D. A. , Garrison, E. P. , Chandler, R. B. , Conner, M. L. , Miller, K. V. , & Cherry, M. J. (2019). Deer movement and resource selection during Hurricane Irma: Implications for extreme climatic events and wildlife. Proceedings of the Royal Society B: Biological Sciences, 286, 20192230. 10.1098/rspb.2019.2230 PMC6939277 31771480

[ece310754-bib-0048] Burch, J. N. (2004). Fire management and resource management at Big Cypress National Preserve. Proceedings of the George Wright Society/National Park Service Joint Conference.

[ece310754-bib-0003] Caudill, G. , & Caudill, D. (2015). Melanism of coyotes (*Canis latrans*) in Florida. The American Midland Naturalist, 174, 335–342.

[ece310754-bib-0004] Cerreta, A. L. , McCarthy, K. P. , & Fowles, G. (2023). Habitat suitability and landscape connectivity for an expanding population of bobcats. Landscape Ecology, 38, 1571–1589.

[ece310754-bib-0005] Cherry, M. J. , Chandler, R. B. , Garrison, E. P. , Crawford, D. A. , Kelly, B. D. , Shindle, D. B. , Godsea, K. G. , Miller, K. V. , & Conner, L. M. (2018). Wildfire affects space use and movement of white‐tailed deer in a tropical pyric landscape. Forest Ecology and Management, 409, 161–169.

[ece310754-bib-0006] Chimney, M. J. , & Goforth, G. (2001). Environmental impacts to the Everglades ecosystem: A historical perspective and restoration strategies. Water Science and Technology, 44, 93–100. 11804164

[ece310754-bib-0049] Crawford, D. A. , Cherry, M. J. , Kelly, B. D. , Garrison, E. P. , Shindle, D. B. , Conner, L. M. , Chandler, R. B. , & Miller, K. V. (2019). Chronology of reproductive investment determines predation risk aversion in a felid‐ungulate system. Ecology and Evolution, 9, 3264–3275. 30962891 10.1002/ece3.4947 PMC6434540

[ece310754-bib-0007] da Silva, L. G. , de Oliveira, T. G. , Kasper, C. B. , Cherem, J. J. , Moraes, E. A., Jr. , Paviolo, A. , & Eizirik, E. (2016). Biogeography of polymorphic phenotypes: Mapping and ecological modelling of coat colour variants in an elusive Neotropical cat, the jaguarundi (*Puma yagouaroundi*). Journal of Zoology, 299, 295–303.

[ece310754-bib-0008] da Silva, L. G. , Kawanishi, K. , Henschel, P. , Kittle, A. , Sanei, A. , Reebin, A. , Miquelle, D. , Stein, A. B. , Watson, A. , Kekule, L. B. , Machado, R. B. , & Eizirik, E. (2017). Mapping black panthers: Macroecological modeling of melanism in leopards (*Panthera pardus*). PLoS One, 12, e0170378. 28379961 10.1371/journal.pone.0170378 PMC5381760

[ece310754-bib-0009] Delhey, K. (2017). Darker where cold and wet: Australian birds follow their own version of Gloger's rule. Ecography, 41, 673–683.

[ece310754-bib-0010] Delhey, K. (2019). A review of Gloger's rule, an ecogeographical rule of colour: Definitions, interpretations and evidence. Biological Reviews, 94, 1294–1316. 30892802 10.1111/brv.12503

[ece310754-bib-0011] Eizirik, E. , Yuhki, N. , Johnson, W. E. , Menotti‐Raymond, M. , Hannah, S. S. , & O'Brien, S. J. (2003). Molecular genetics and evolution of melanism in the cat family. Current Biology, 13, 448–453. 12620197 10.1016/s0960-9822(03)00128-3

[ece310754-bib-0012] Forsman, A. , Karlsson, M. , Wennersten, L. , Johansson, J. , & Karpestam, E. (2011). Rapid evolution of fire melanism in replicated populations of pygmy grasshoppers. Evolution, 65, 2530–2540. 21884054 10.1111/j.1558-5646.2011.01324.x

[ece310754-bib-0013] Graipel, M. E. , Bogoni, J. A. , Giehl, E. L. H. , Cerezer, F. O. , Cáceres, N. C. , & Eizirik, E. (2019). Melanism evolution in the cat family is influenced by intraspecific communication under low visibility. PLoS One, 14, e0226136. 31851714 10.1371/journal.pone.0226136 PMC6919575

[ece310754-bib-0014] Graipel, M. E. , Oliveira‐Santos, L. G. R. , Goulart, F. V. B. , Tortato, M. A. , Miller, P. R. M. , & Cáceres, N. C. (2014). The role of melanism in oncillas on the temporal segregation of nocturnal activity. Brazilian Journal of Biology, 74, 142–145. 10.1590/1519-6984.14312 25627377

[ece310754-bib-0015] Guthrie, R. D. (1967). Fire melanism among mammals. The American Midland Naturalist, 77, 227–230.

[ece310754-bib-0016] Guyette, R. P. , Stambaugh, M. C. , Dey, D. C. , & Muzika, R. (2012). Predicting fire frequency with chemistry and climate. Ecosystems, 15, 322–335.

[ece310754-bib-0017] Hocking, B. (1964). Fire melanism in some African grasshoppers. Evolution, 18, 332–335.

[ece310754-bib-0018] Hutchinson, J. T. , & Hutchinson, T. (2000). Observation of a melanistic bobcat in the Ocala National Forest. Florida Field Naturalist, 28, 25–26.

[ece310754-bib-0019] James, F. C. (1991). Complementary descriptive and experimental studies of clinal variation in birds. American Zoologist, 31, 694–706.

[ece310754-bib-0020] Johnson, W. E. , Onorato, D. P. , Roelke, M. E. , Land, E. D. , Cunningham, M. , Belden, R. C. , McBride, R. , Jansen, D. , Lotz, M. , Shindle, D. , Howard, J. , Wildt, D. E. , Penfold, L. M. , Hostetler, J. A. , Oli, M. K. , & O'Brien, S. J. (2010). Genetic restoration of the Florida panther. Science, 329, 1641–1645. 20929847 10.1126/science.1192891 PMC6993177

[ece310754-bib-0021] Karlsson, M. , Caesar, S. , Ahnesjö, J. , & Forsman, A. (2008). Dynamics of colour polymorphism in a changing environment: Fire melanism and then what? Oecologia, 154, 715–724. 17957385 10.1007/s00442-007-0876-y

[ece310754-bib-0022] Kiltie, R. A. (1989). Wildfire and the evolution of dorsal melanism in fox squirrels, *Sciurus niger* . Journal of Mammalogy, 70, 726–739.

[ece310754-bib-0023] Kingsley, E. P. , Manceau, M. , Wiley, C. D. , & Hoekstra, H. E. (2009). Melanism in *Peromyscus* is caused by independent mutations in agouti. PLoS One, 4, e6435. 19649329 10.1371/journal.pone.0006435 PMC2713407

[ece310754-bib-0024] Kitchener, A. C. , Breitenmoser‐Würsten, C. , Eizirik, E. , Gentry, A. , Werdelin, L. , Wilting, A. , Yamaguchi, N. , Abramov, A. V. , Christiansen, P. , Driscoll, C. , Duckworth, J. W. , Johnson, W. E. , Luo, S. J. , Meijaard, E. , O'Donoghue, P. , Sanderson, J. , Seymour, K. , Bruford, M. , Groves, C. , … Tobe, S. (2017). A revised taxonomy of the Felidae: The final report of the Cat Classification Task Force of the IUCN Cat Specialist Group .

[ece310754-bib-0025] Kominoski, J. S. , Fernandez, M. , Breault, P. , Sclater, V. , & Rothermel, B. B. (2022). Fire severity and post‐fire hydrology drive nutrient cycling and plant community recovery in intermittent wetlands. Ecosystems, 25, 265–278.

[ece310754-bib-0026] Lavariega, M. C. , Briones‐Salas, M. , Monroy‐Gamboa, A. G. , & Ramos‐Mendez, D. (2022). Population density and daily activity patterns of bobcats in its southernmost continental distribution. Animal Biodiversity and Conservation, 45(2), 145–160.

[ece310754-bib-0027] Lockwood, J. L. , Ross, M. S. , & Sah, J. P. (2003). Smoke on the water: The interplay of fire and water flow on Everglades restoration. Frontiers in Ecology and the Environment, 1, 462–468.

[ece310754-bib-0028] Main, M. B. , & Richardson, L. W. (2002). Response of wildlife to prescribed fire in Southwest Florida pine flatwoods. Wildlife Society Bulletin, 30, 213–221.

[ece310754-bib-0029] Mayr, E. (1963). Animal species and evolution. Harvard University Press.

[ece310754-bib-0030] McAlpine, D. (2021). Further occurrences of melanism in a northern, peripheral, population of bobcat (*Lynx rufus*). The Canadian Field‐Naturalist, 135, 52–57.

[ece310754-bib-0031] Mooring, M. S. , Eppert, A. A. , & Botts, R. T. (2020). Natural selection of melanism in costa Rican jaguar and oncilla: A test of Gloger's rule and the temporal segregation hypothesis. Tropical Conservation Science, 13, 194008292091036.

[ece310754-bib-0032] Nachman, M. W. , Hoekstra, H. E. , & D'Agostino, S. L. (2003). The genetic basis of adaptive melanism in pocket mice. Proceedings of the National Academy of Sciences, 100, 5268–5273. 10.1073/pnas.0431157100 PMC154334 12704245

[ece310754-bib-0033] Pike, J. R. , Shaw, J. H. , Leslie, D. M. , & Shaw, M. G. (1999). A geographic analysis of the status of mountain lions in Oklahoma. Wildlife Society Bulletin, 27, 4–11.

[ece310754-bib-0034] Potash, A. D. , Greene, D. U. , Mathis, V. L. , Baiser, B. , Conner, L. M. , & McCleery, R. A. (2020). Ecological drivers of eastern fox squirrel pelage polymorphism. Frontiers in Ecology and Evolution, 8, 1–9.

[ece310754-bib-0035] Regan, T. W. , & Maer, D. S. (1990). Melanistic bobcats in Florida. Florida Field Naturalist, 18, 84–87.

[ece310754-bib-0036] Reissmann, M. , Lutz, W. , Lieckfeldt, D. , Sandoval‐Castellanos, E. , & Ludwig, A. (2020). An agouti‐signaling‐protein mutation is strongly associated with melanism in European roe deer (*Capreolus capreolus*). Genes, 11, 647. 32545389 10.3390/genes11060647 PMC7349051

[ece310754-bib-0037] Rising, J. D. , Jackson, D. A. , & Fokidis, H. B. (2009). Geographic variation in plumage pattern and coloration of savannah sparrows. The Wilson Journal of Ornithology, 121, 253–264.

[ece310754-bib-0038] Roberts, N. M. , & Crimmins, S. M. (2010). Bobcat population status and management in North America: Evidence of large‐scale population increase. Journal of Fish and Wildlife Management, 1, 169–174.

[ece310754-bib-0039] Rosatte, R. (2011). Evidence confirms the presence of cougars (*Puma concolor*) in Ontario, Canada. The Canadian Field‐Naturalist, 125, 116–125.

[ece310754-bib-0040] Ruiz, P. L. , Sah, J. P. , Ross, M. S. , & Spitzig, A. A. (2013). Tree Island response to fire and flooding in the short‐hydroperiod marl prairie grasslands of the Florida Everglades, USA. Fire Ecology, 9, 38–54.

[ece310754-bib-0041] Schneider, A. , Henegar, C. , Day, K. , Absher, D. , Napolitano, C. , Silveira, L. , David, V. A. , O'Brien, S. J. , Menotti‐Raymond, M. , Barsh, G. S. , & Eizirik, E. (2015). Recurrent evolution of melanism in south American felids. PLoS Genetics, 11, e1004892. 25695801 10.1371/journal.pgen.1004892 PMC4335015

[ece310754-bib-0042] Slocum, M. G. , Platt, W. J. , & Cooley, H. C. (2003). Effects of differences in prescribed fire regimes on patchiness and intensity of fires in subtropical savannas of Everglades National Park, Florida. Restoration Ecology, 11, 91–102.

[ece310754-bib-0043] Smith, T. J. , Foster, A. M. , Tiling‐Range, G. , & Jones, J. W. (2013). Dynamics of mangrove‐marsh ecotones in subtropical coastal wetlands: Fire, sea‐level rise, and water levels. Fire Ecology, 9, 66–77.

[ece310754-bib-0044] Tischendorf, J. W. , & McAlpine, D. F. (1995). A melanistic bobcat from outside Florida. Florida Field Naturalist, 23, 13–14.

[ece310754-bib-0045] Ulmer, F. A. (1941). Melanism in the Felidae, with special reference to the genus *Lynx* . Journal of Mammalogy, 22, 285–288.

[ece310754-bib-0046] Uy, J. A. C. , Cooper, E. A. , Cutie, S. , Concannon, M. R. , Poelstra, J. W. , Moyle, R. G. , & Filardi, C. E. (2016). Mutations in different pigmentation genes are associated with parallel melanism in Island flycatchers. Proceedings of the Royal Society B: Biological Sciences, 283, 20160731. 10.1098/rspb.2016.0731 PMC4947890 27412275

[ece310754-bib-0047] VanderWerf, E. A. (2012). Ecogeographic patterns of morphological variation in Elepaios (*Chasiempis* spp.): Bergmann's, Allen's, and Gloger's rules in a microcosm – Patrones Ecogeográficos de Variación Morfológica en *Chasiempis* spp.: Las Reglas de Bergmann, Allen y Gloger en un microcosmos. Ornithological Monographs, 73, 1–34.

