# Supplementary material for: Photographic documentation of melanism in bobcats (Lynx rufus) in the Greater Everglades
Source: Ecol Evol. 2024 Jan 16;14(1):e10754. doi: 10.1002/ece3.10754 (PMC10791593; doi:10.1002/ece3.10754)
Supplement: Supplementary file 1 — Appendix S1. [file ECE3-14-e10754-s001.pdf]

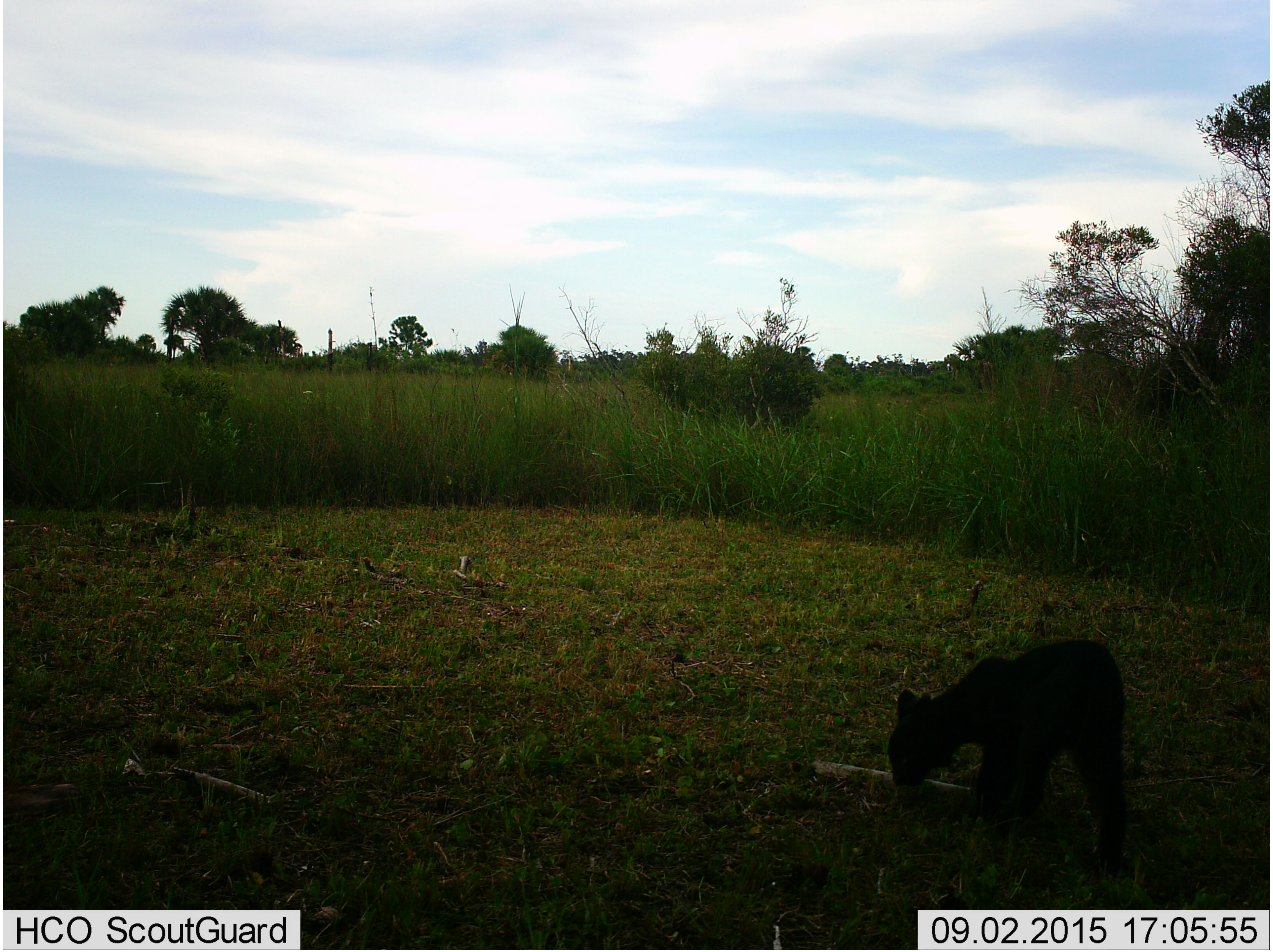
HCO ScoutGuard
09.02.2015 17:05:55

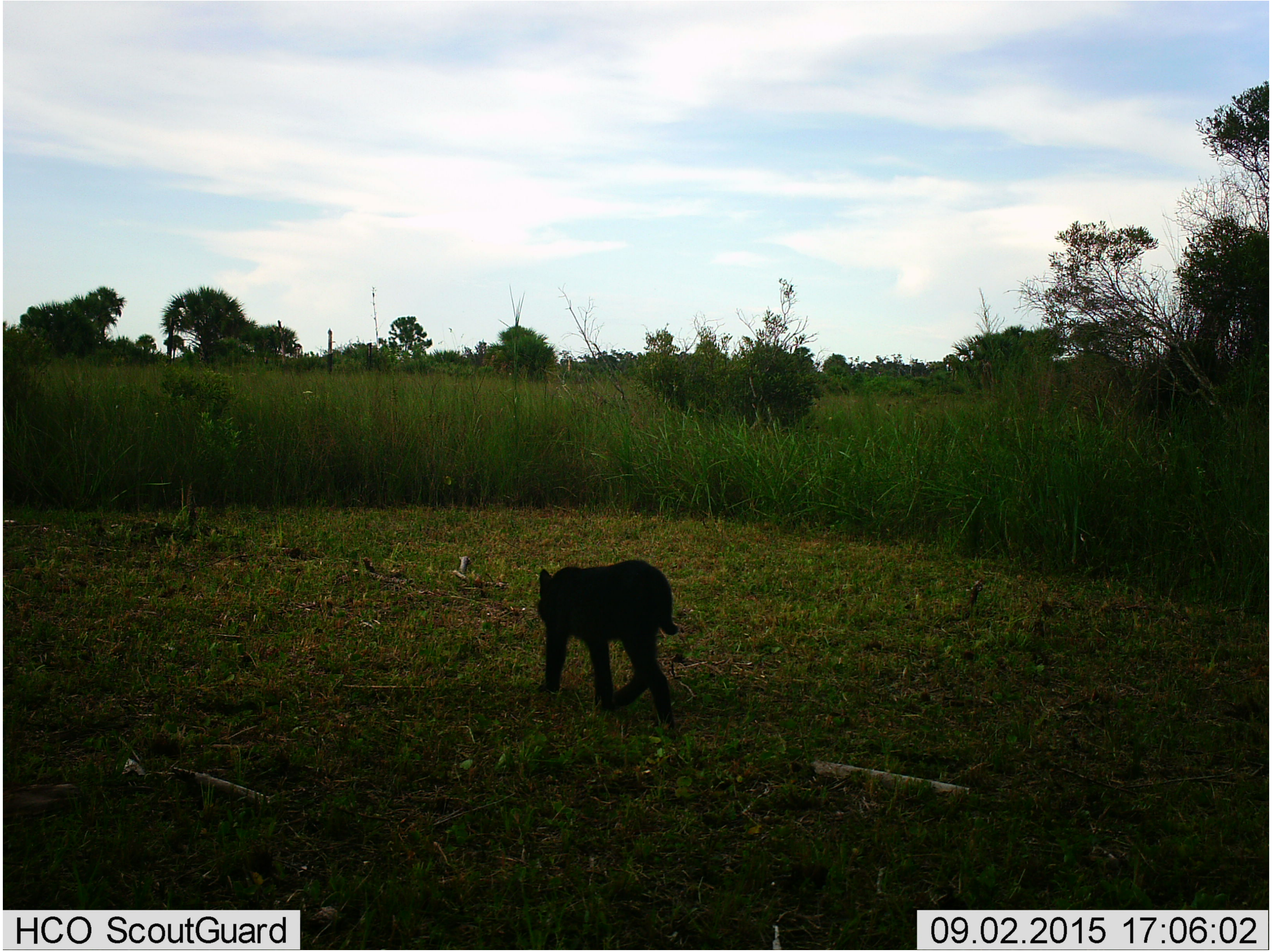
HCO ScoutGuard
09.02.2015 17:06:02

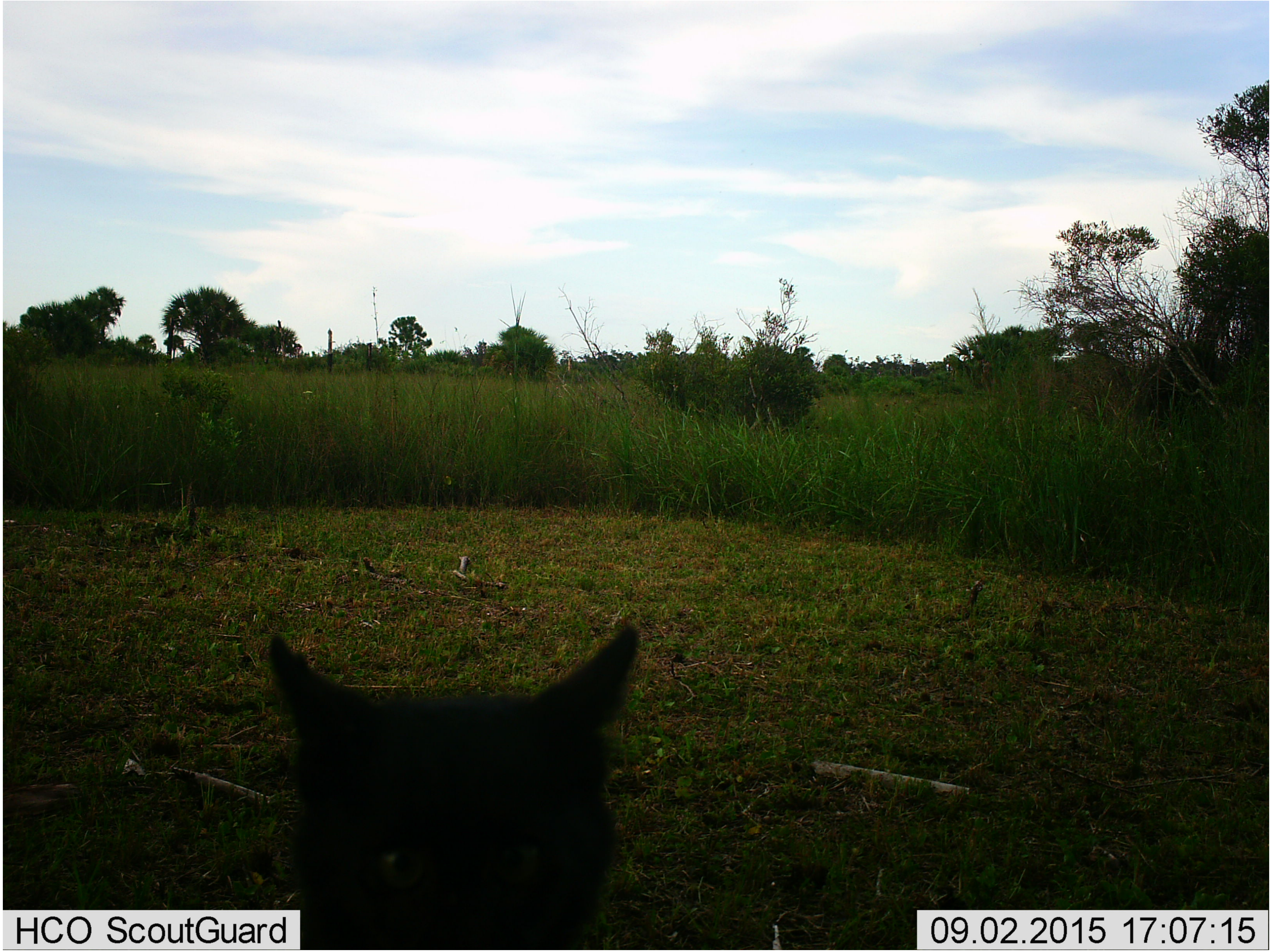
HCO ScoutGuard
09.02.2015 17:07:15

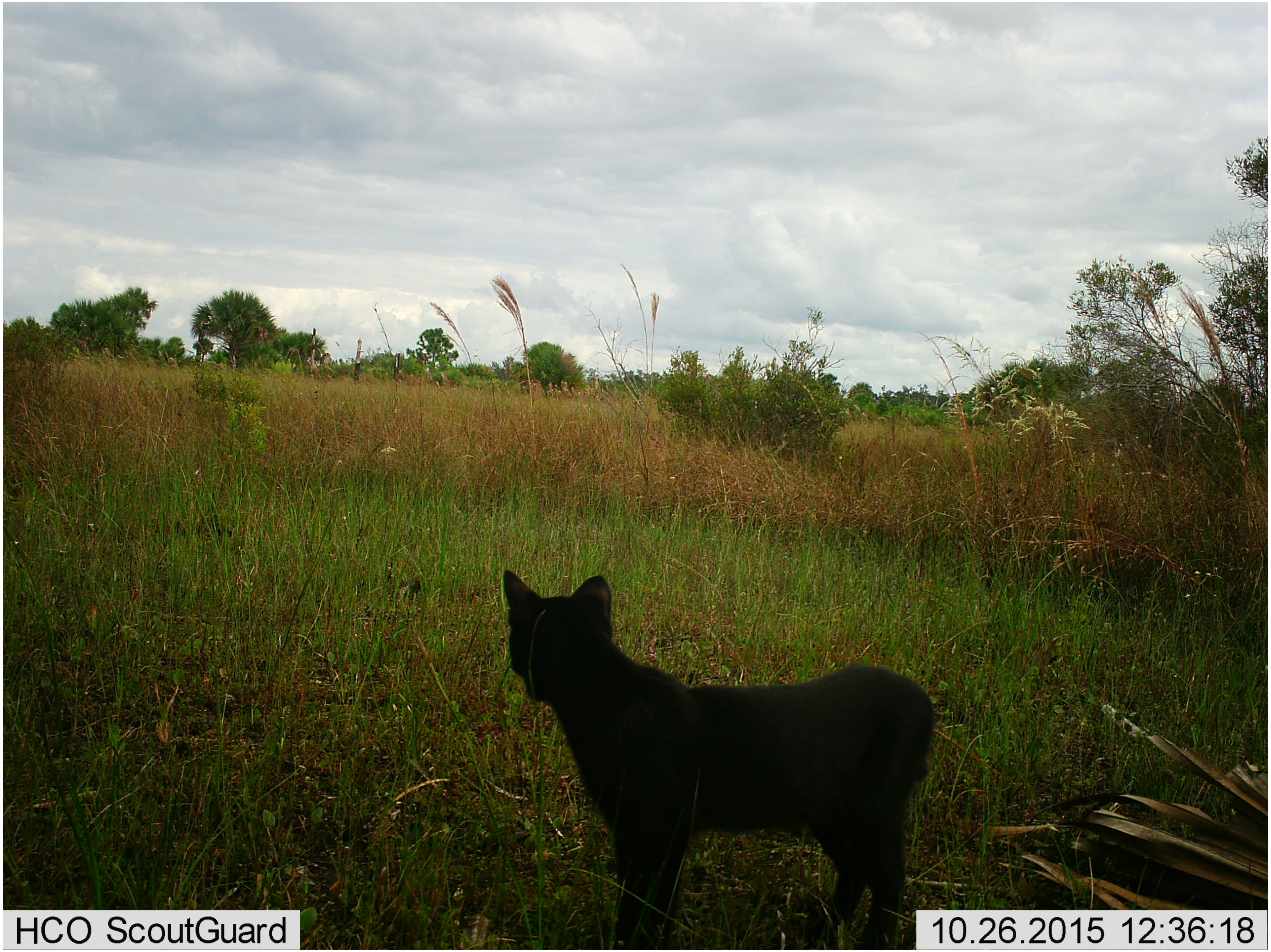
HCO ScoutGuard
10.26.2015 12:36:18

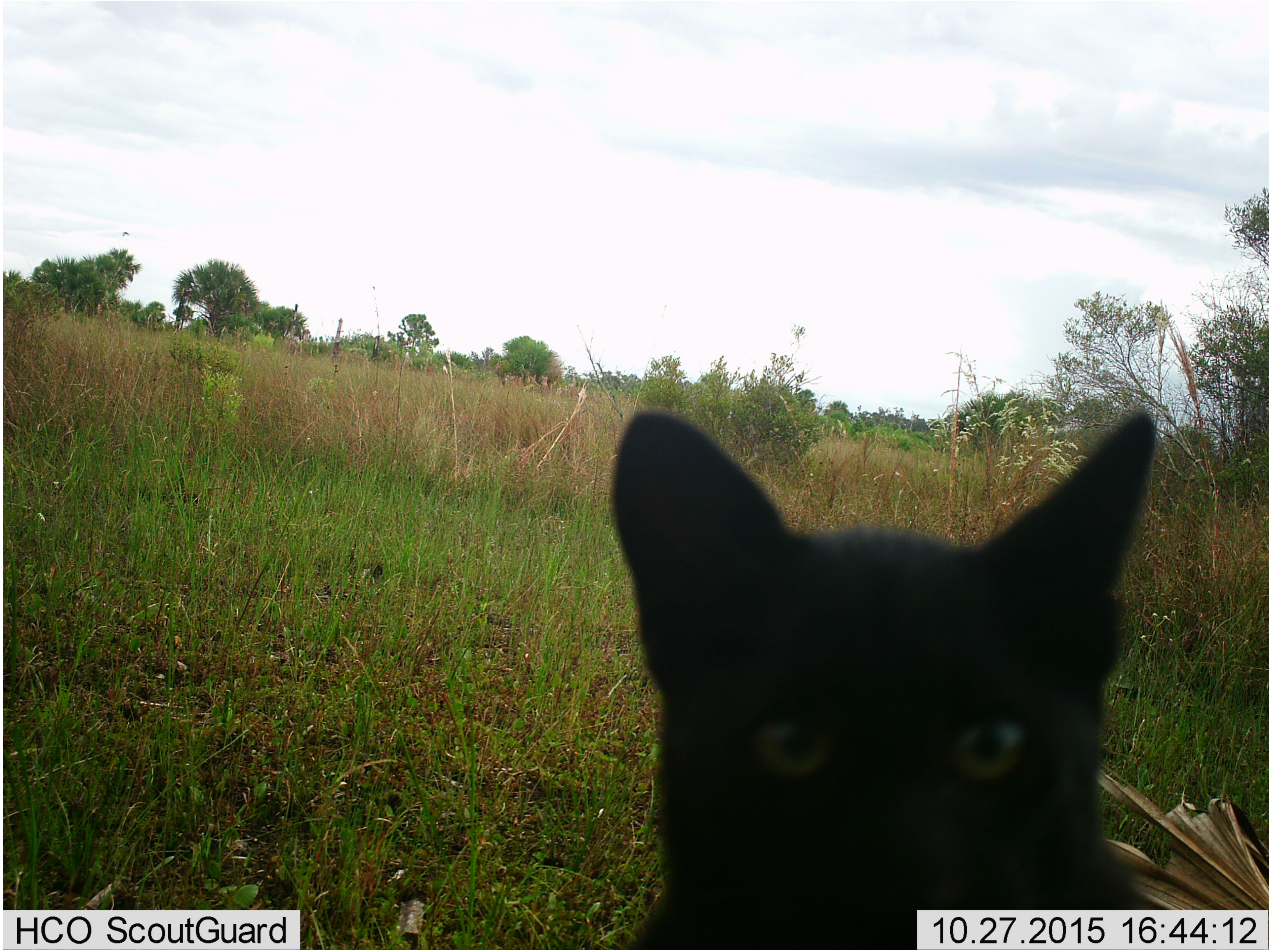
HCO ScoutGuard
10.27.2015 16:44:12

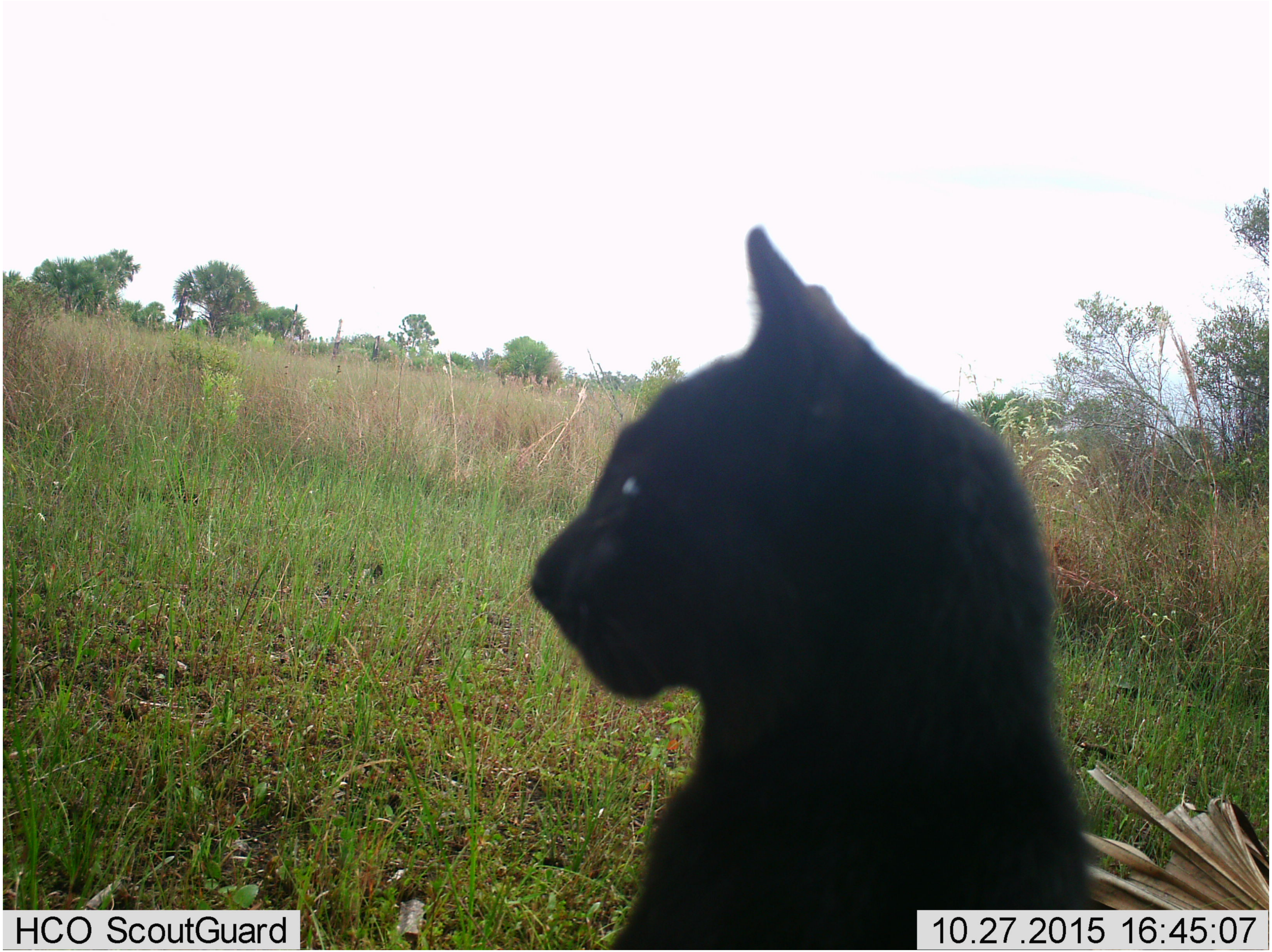
HCO ScoutGuard
10.27.2015 16:45:07

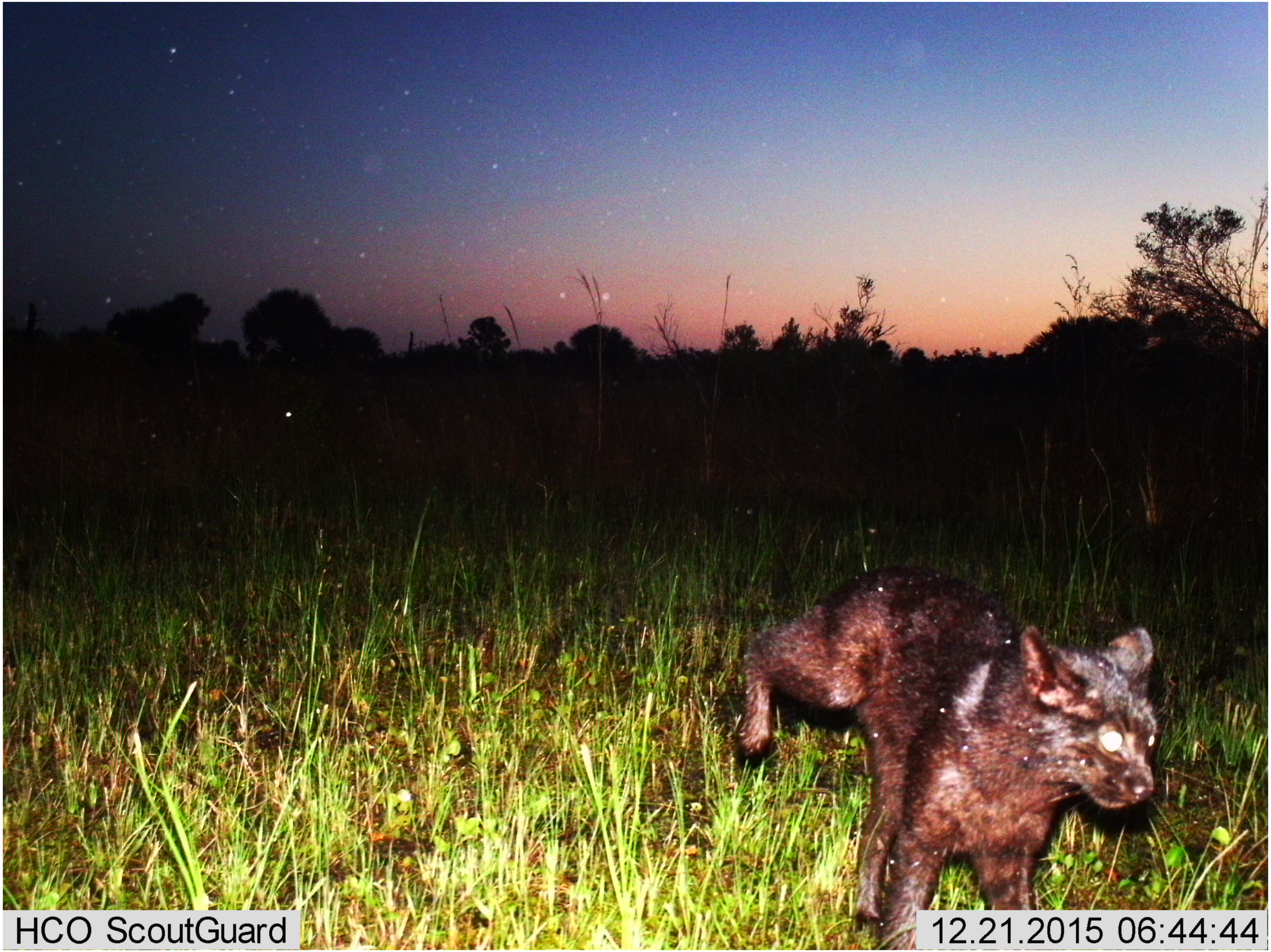
HCO ScoutGuard
12.21.2015 06:44:44

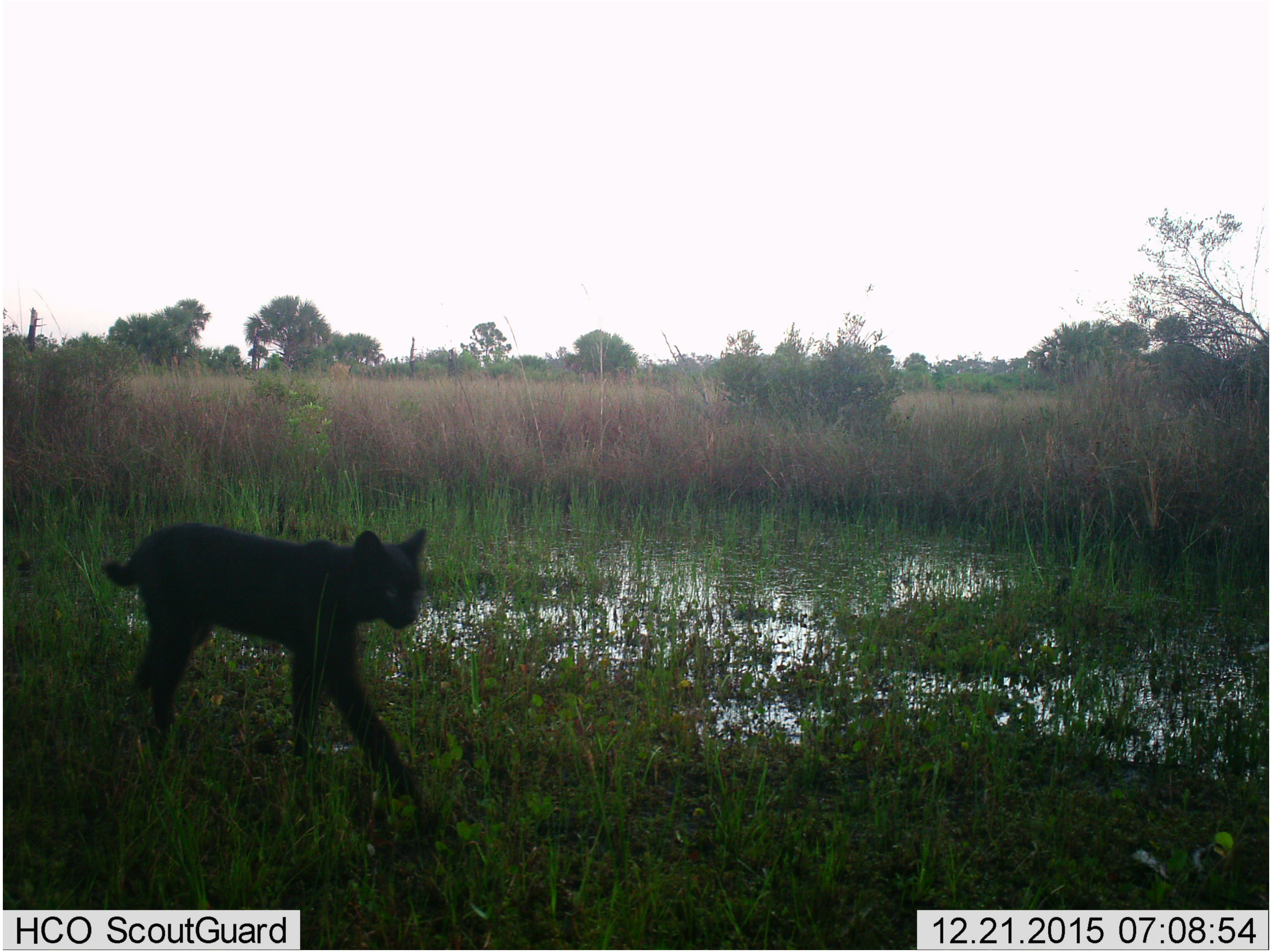
HCO ScoutGuard
12.21.2015 07:08:54

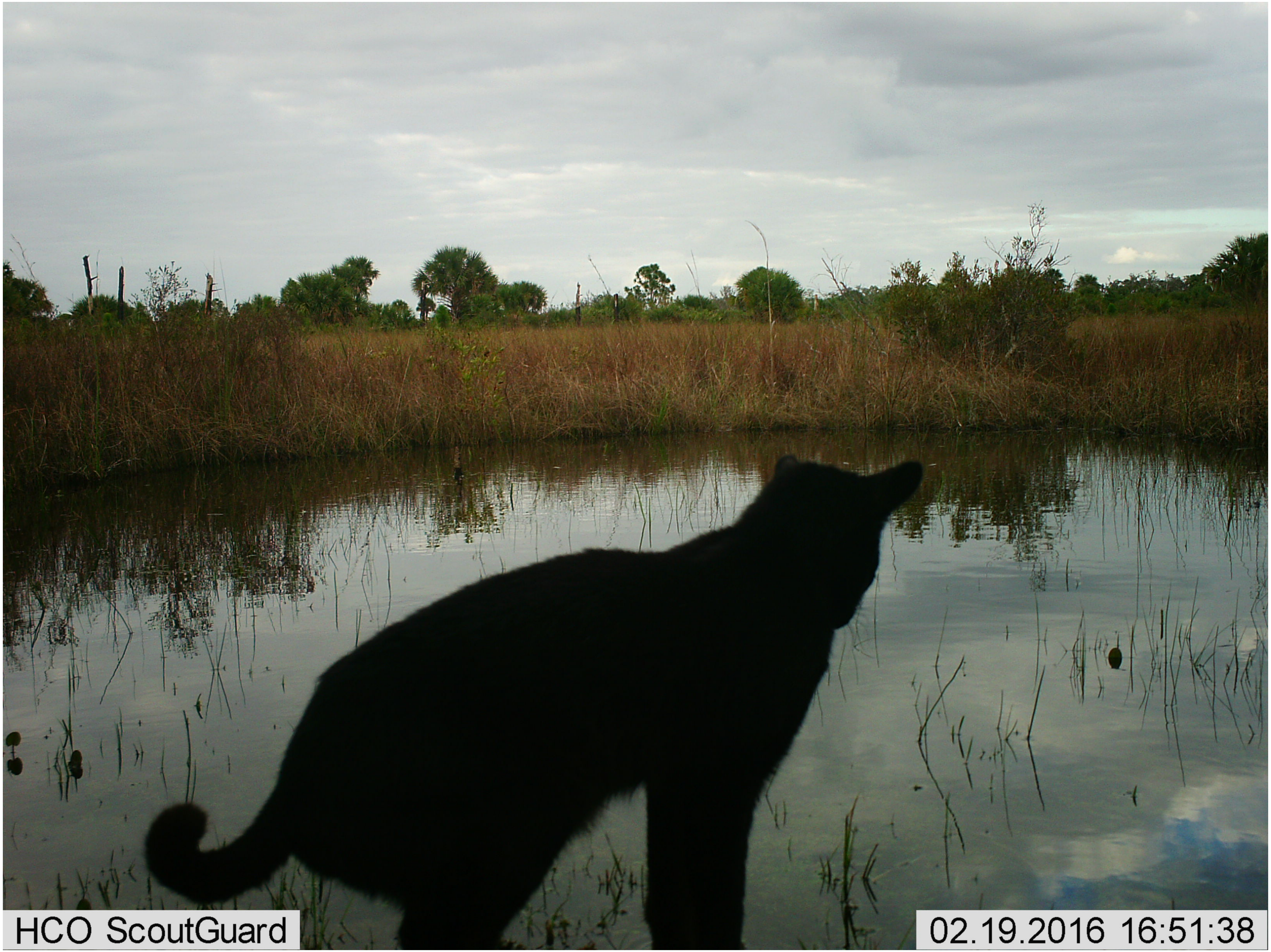
HCO ScoutGuard
02.19.2016 16:51:38

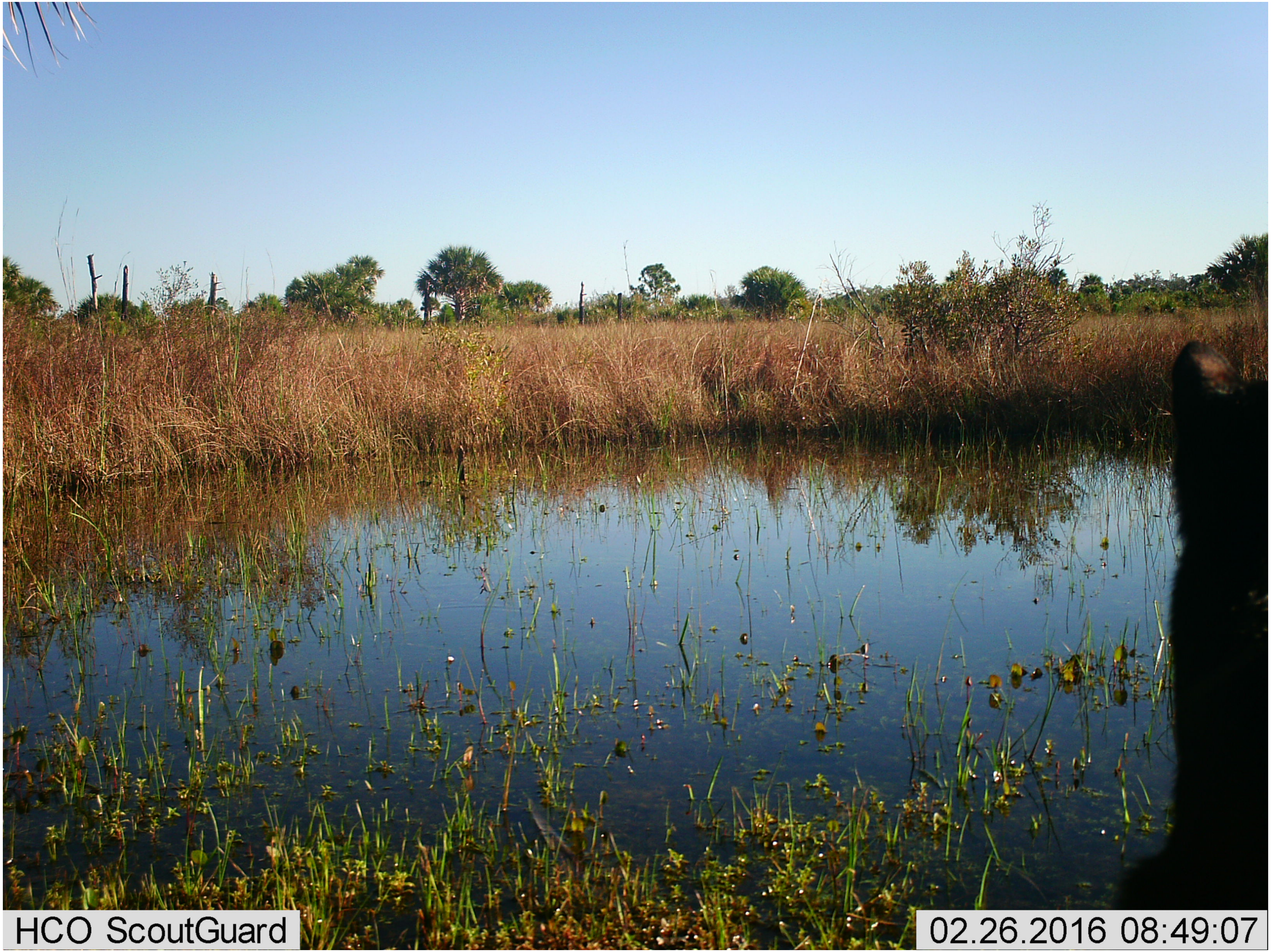
HCO ScoutGuard
02.26.2016 08:49:07

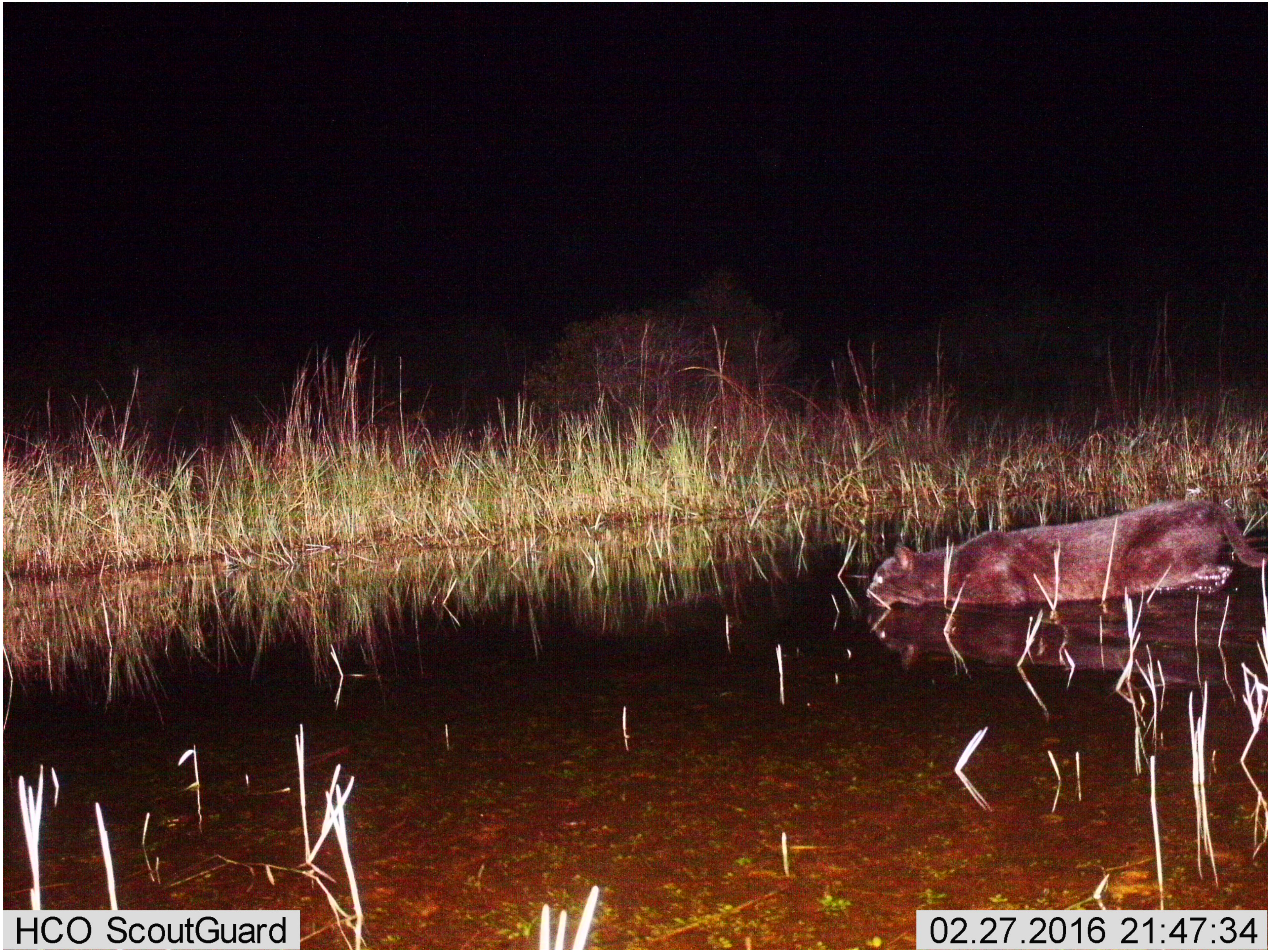
HCO ScoutGuard
02.27.2016 21:47:34

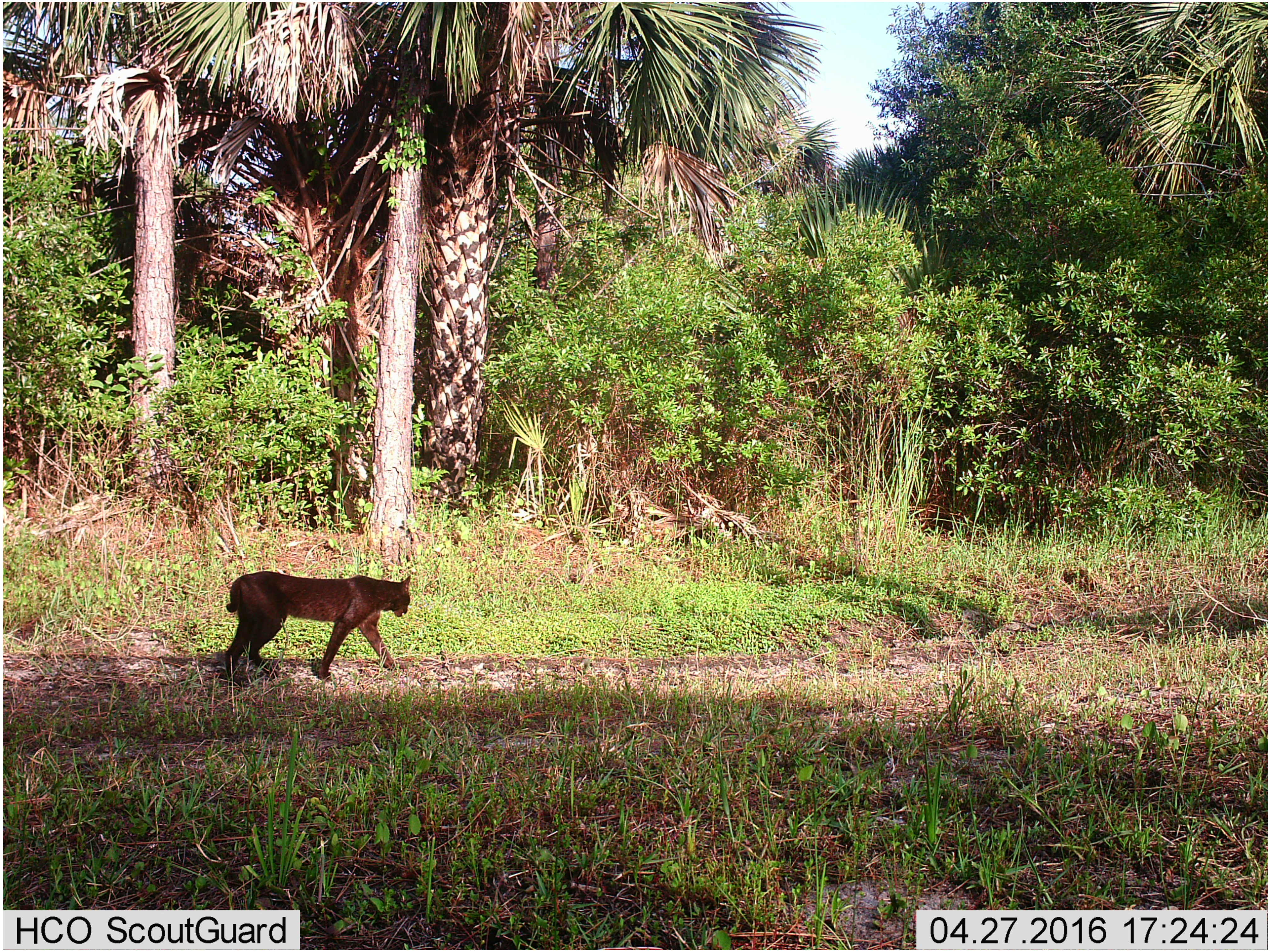
HCO ScoutGuard
04.27.2016 17:24:24

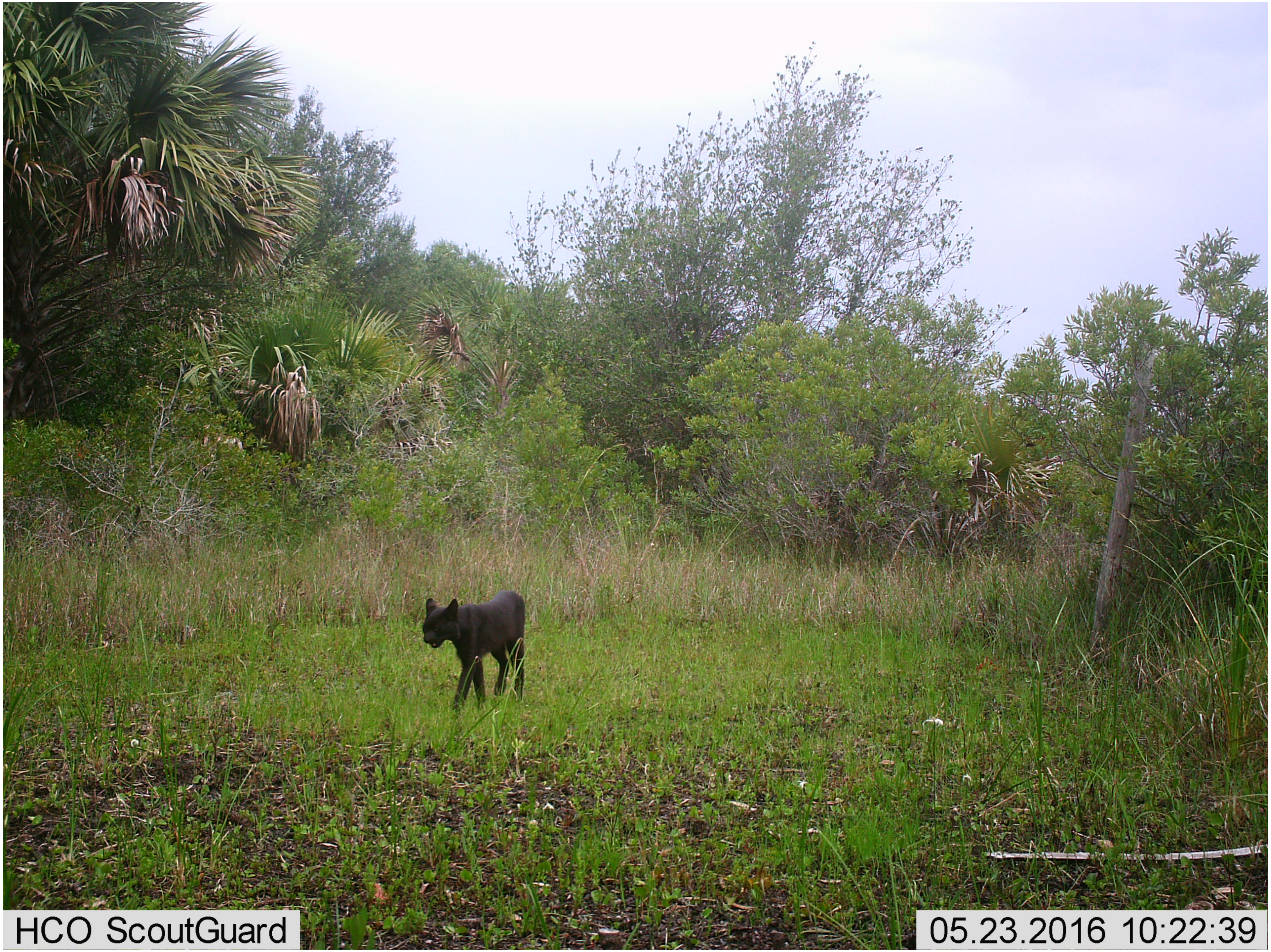
HCO ScoutGuard
05.23.2016 10:22:39

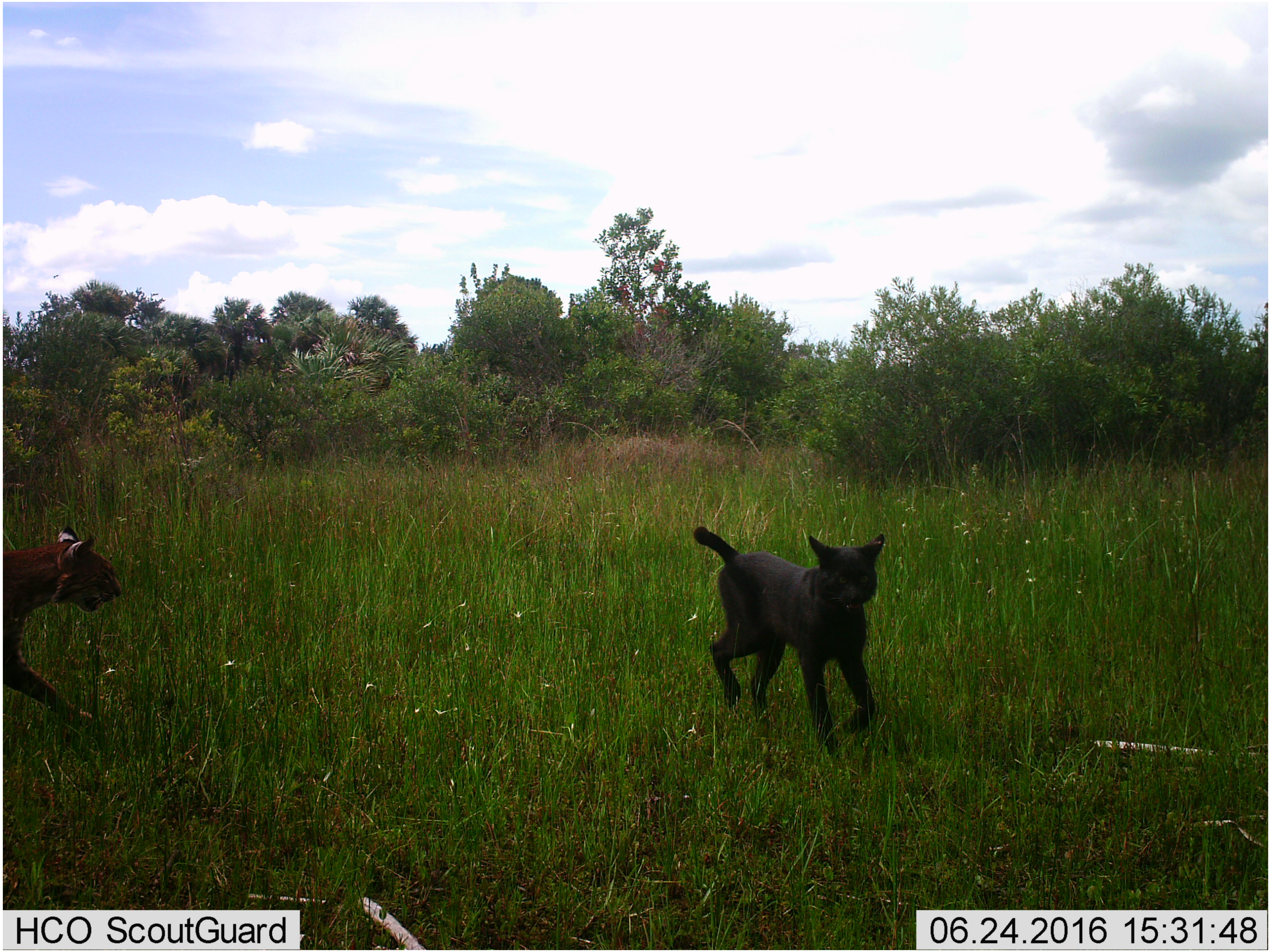
HCO ScoutGuard
06.24.2016 15:31:48

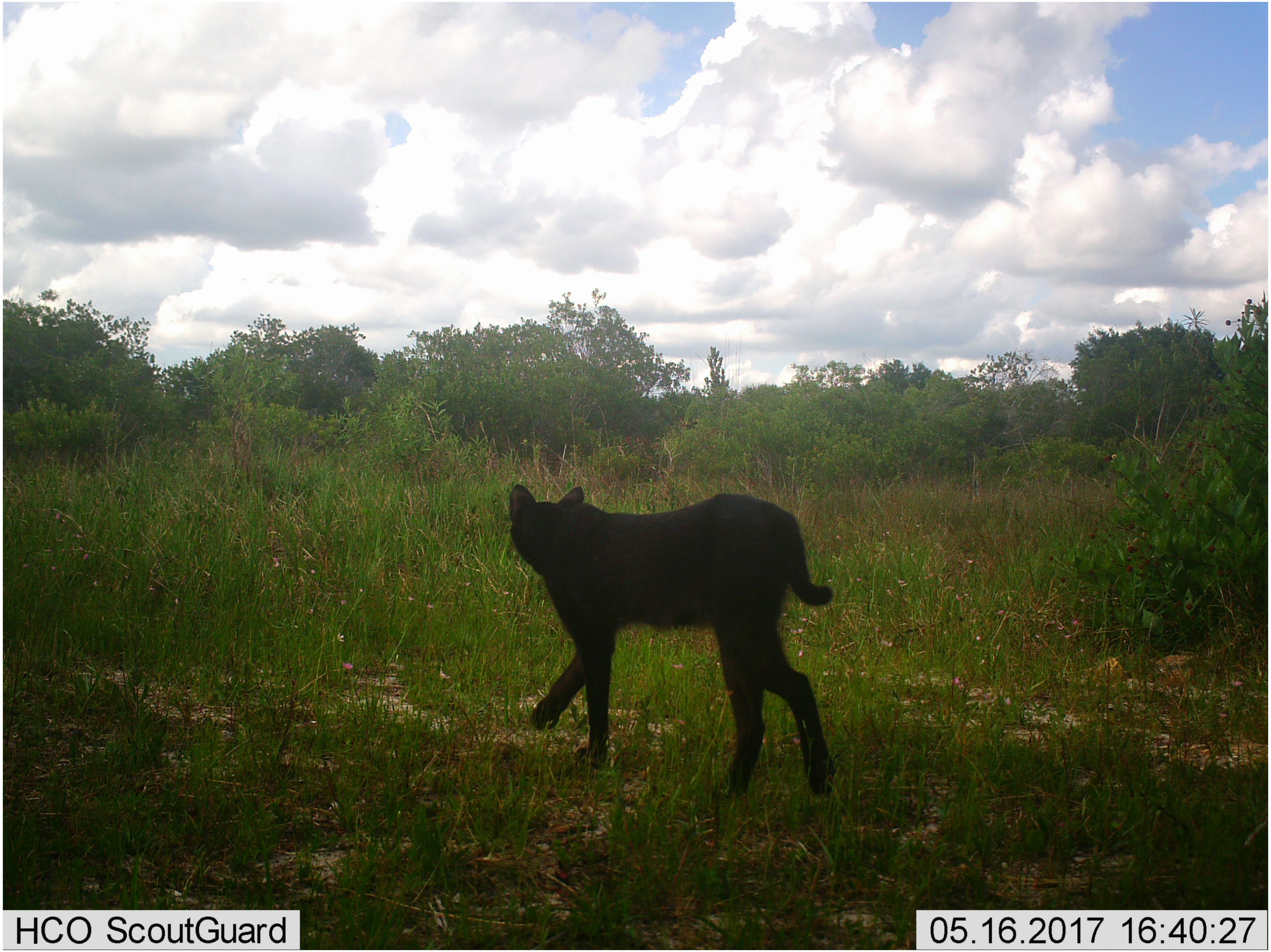
HCO ScoutGuard
05.16.2017 16:40:27

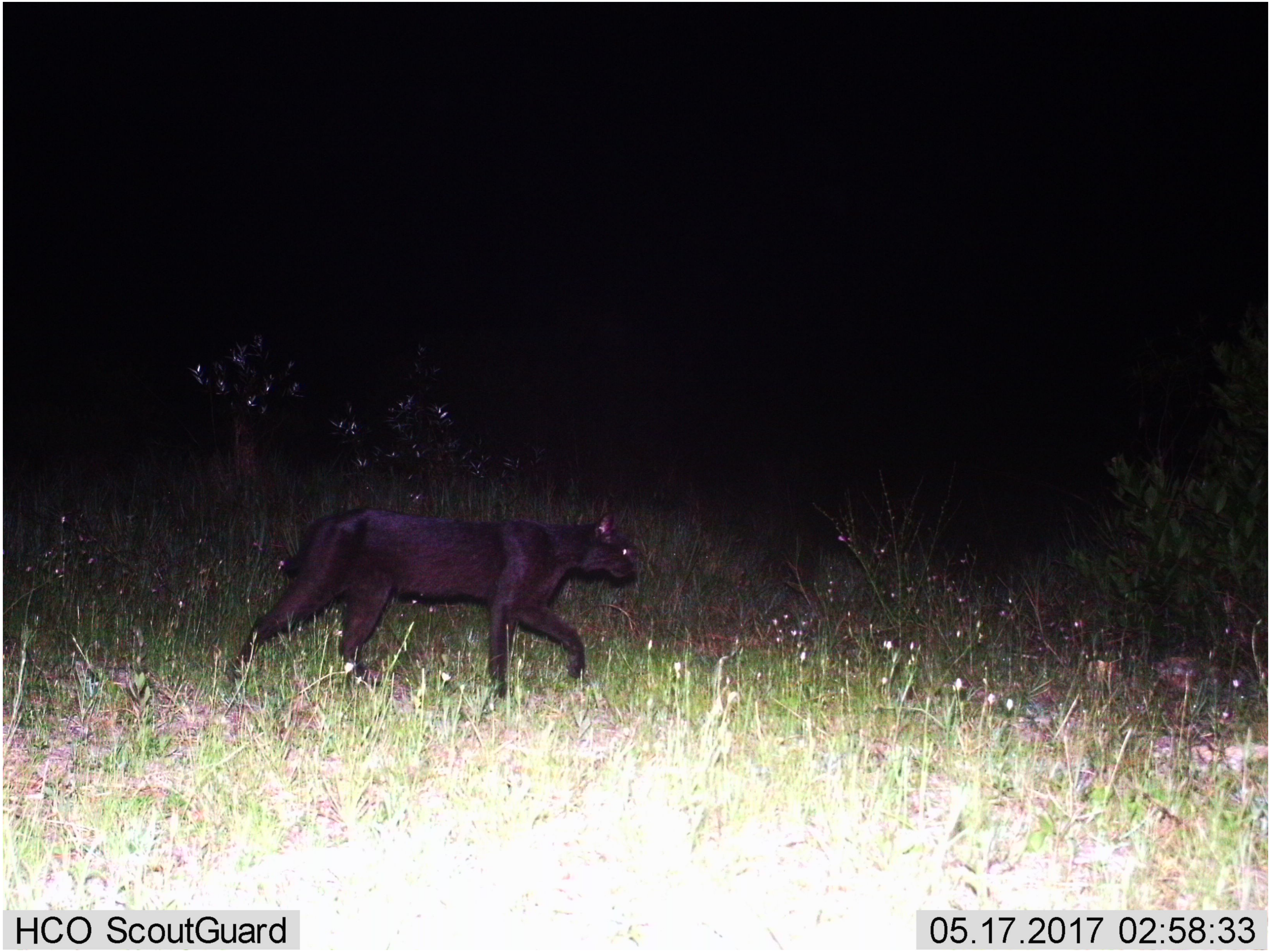
HCO ScoutGuard
05.17.2017 02:58:33

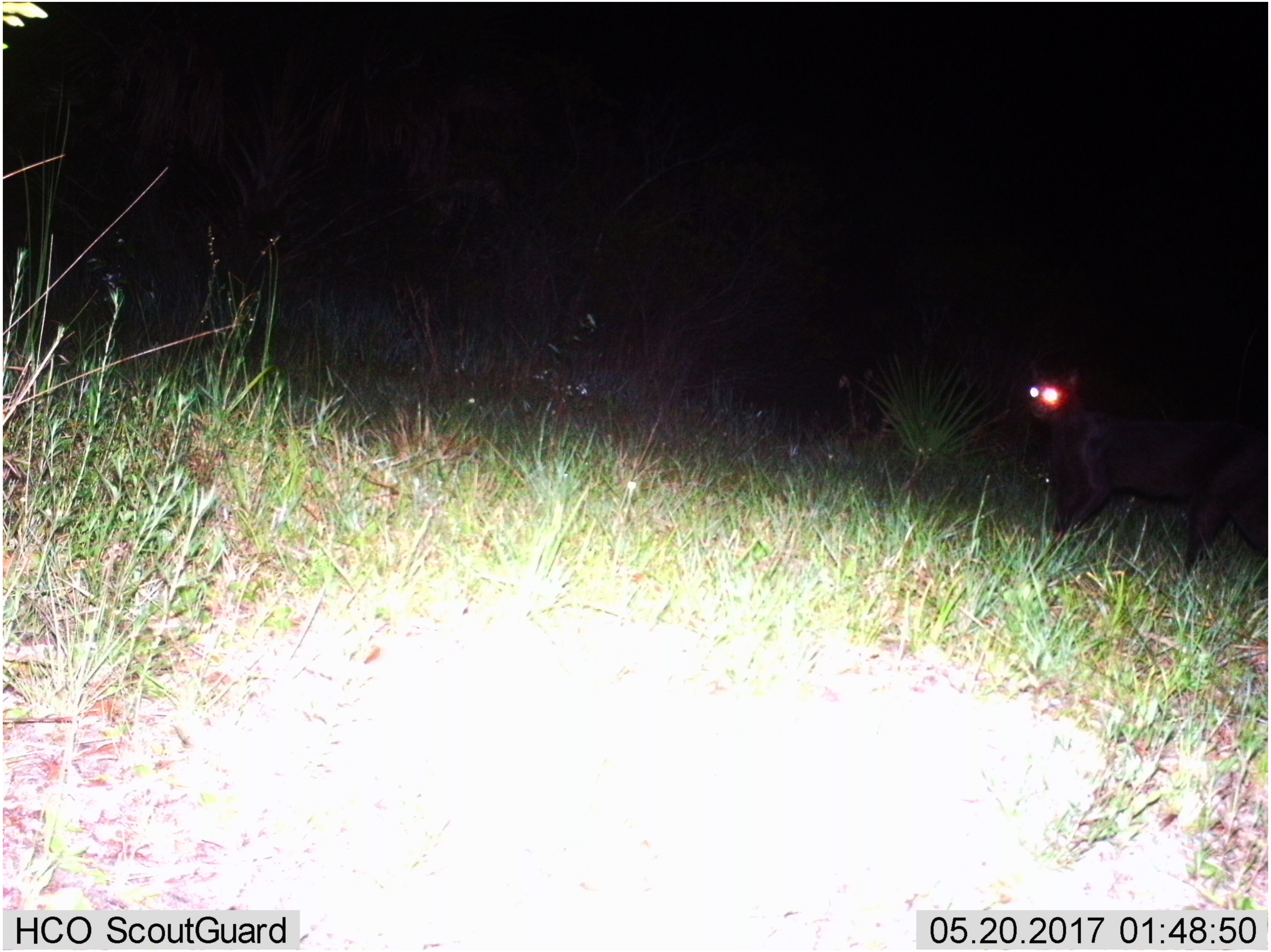
HCO ScoutGuard
05.20.2017 01:48:50

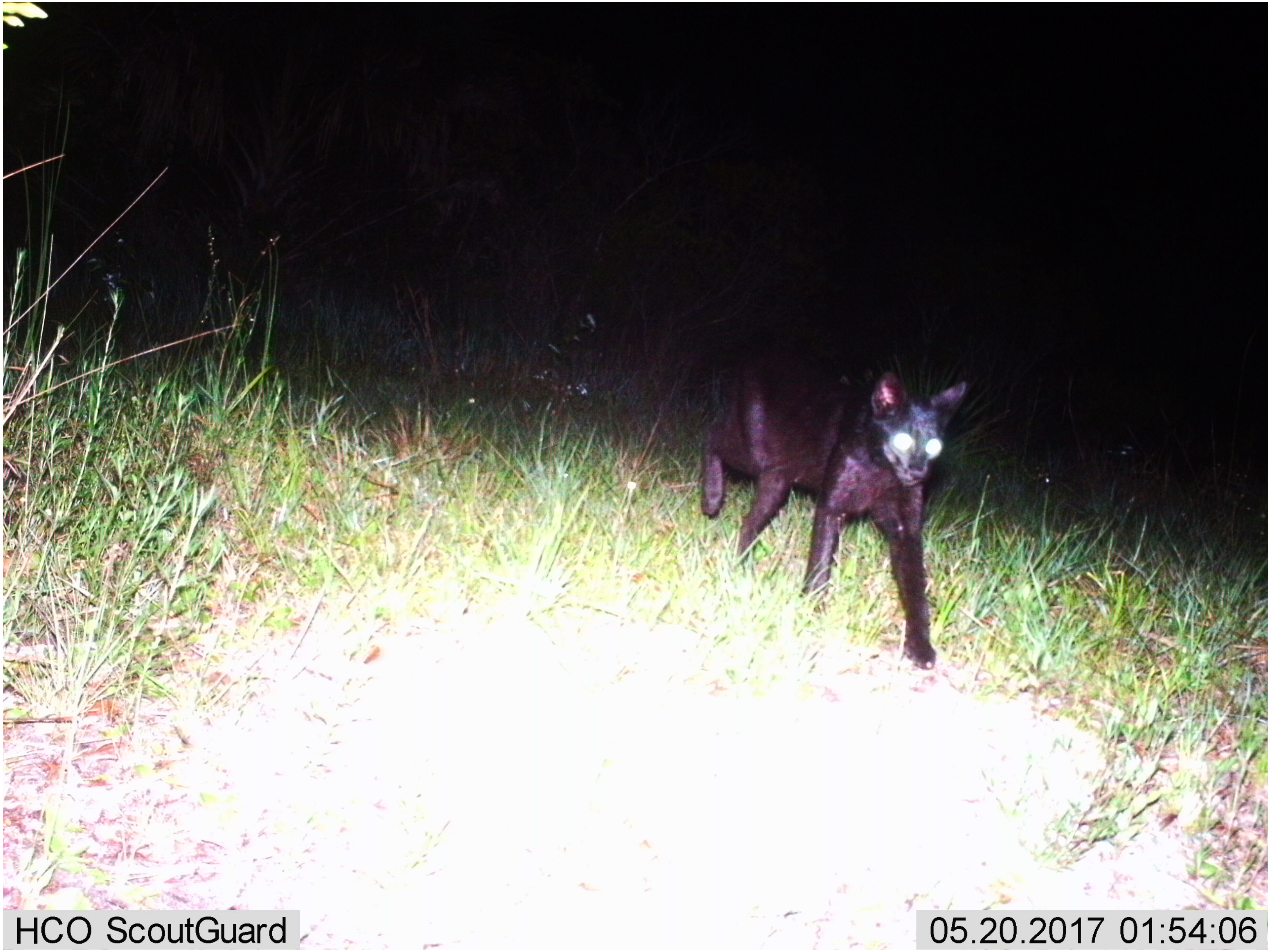
HCO ScoutGuard
05.20.2017 01:54:06

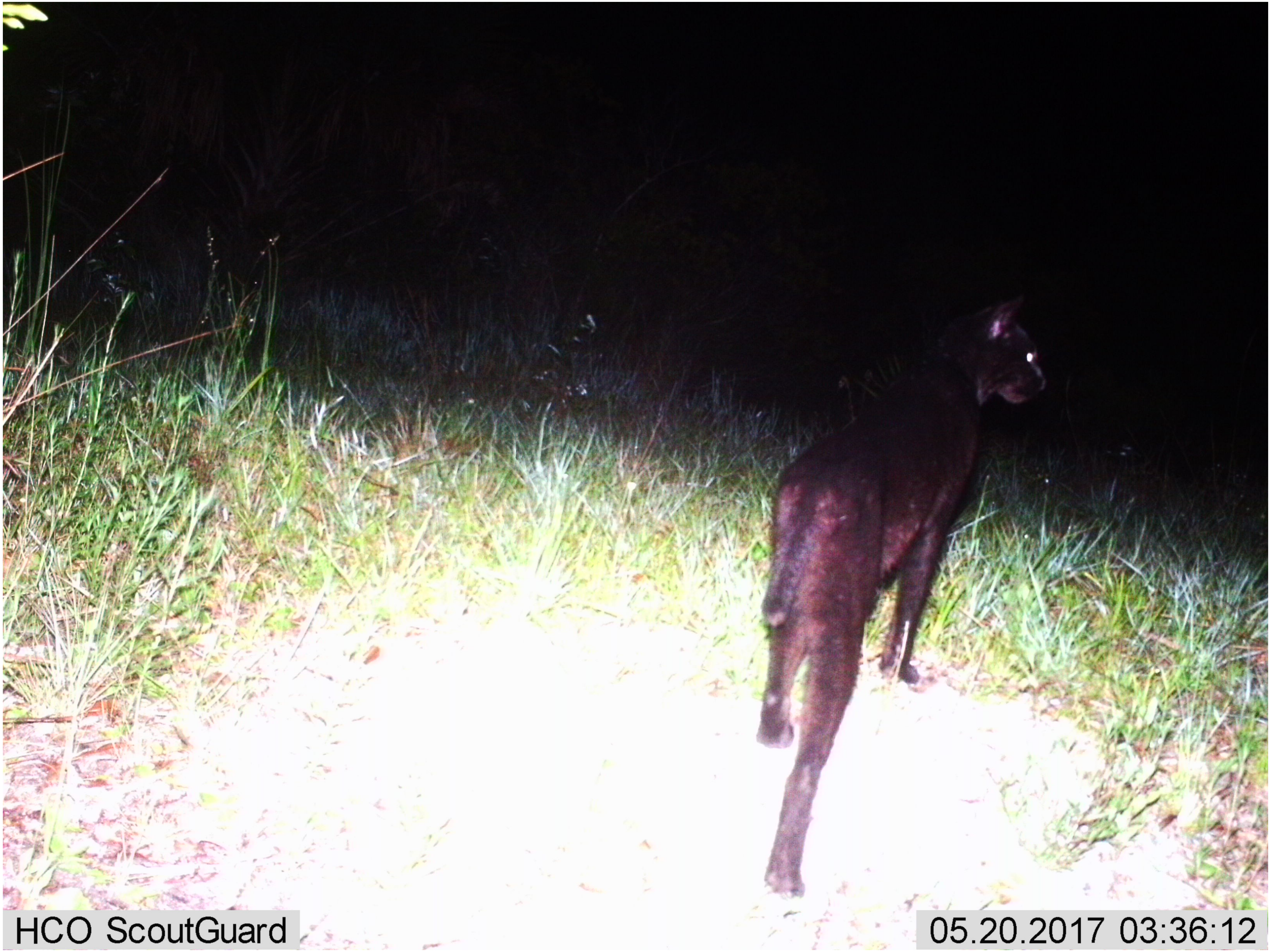
HCO ScoutGuard
05.20.2017 03:36:12

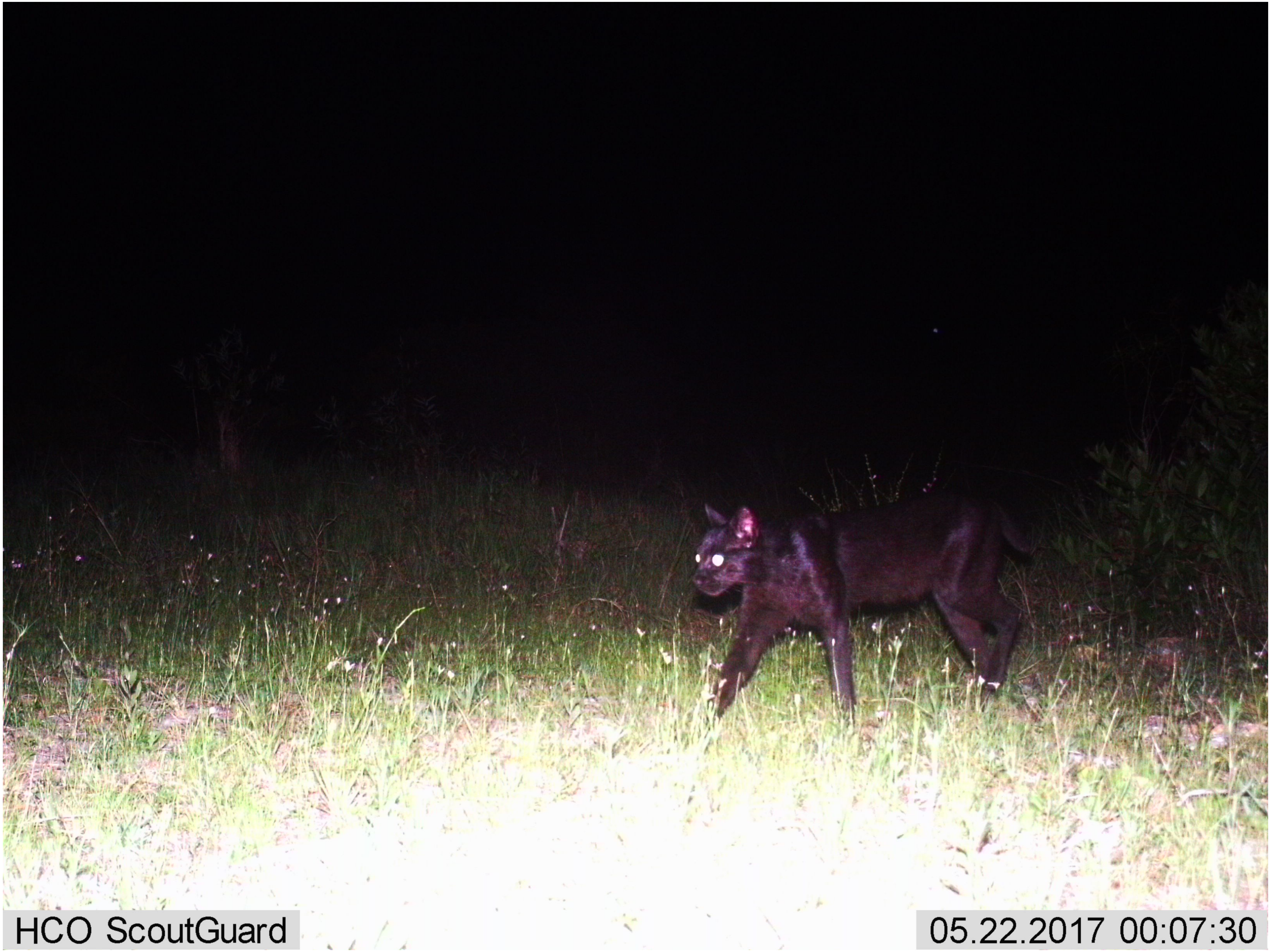
HCO ScoutGuard
05.22.2017 00:07:30

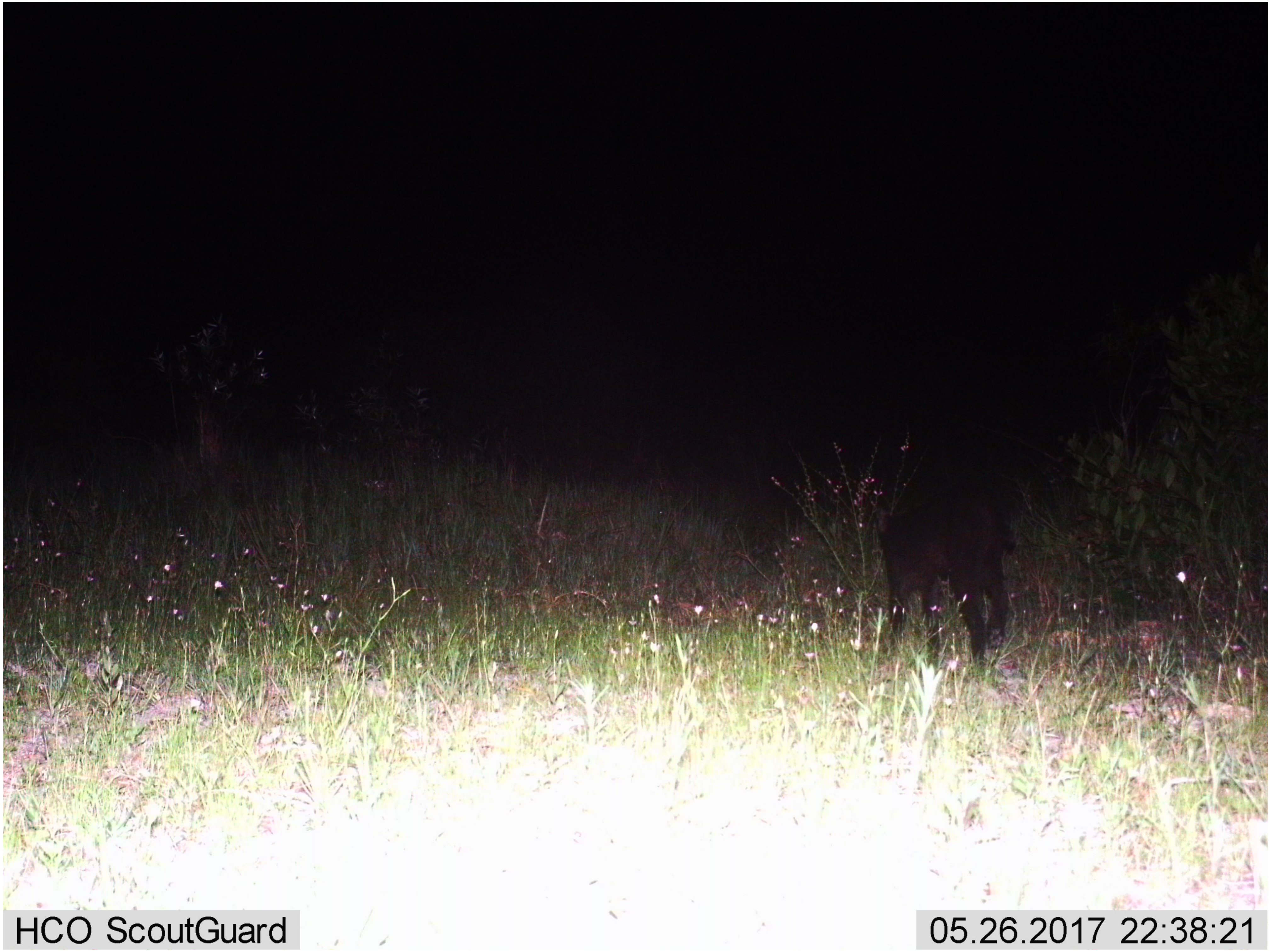
HCO ScoutGuard
05.26.2017 22:38:21

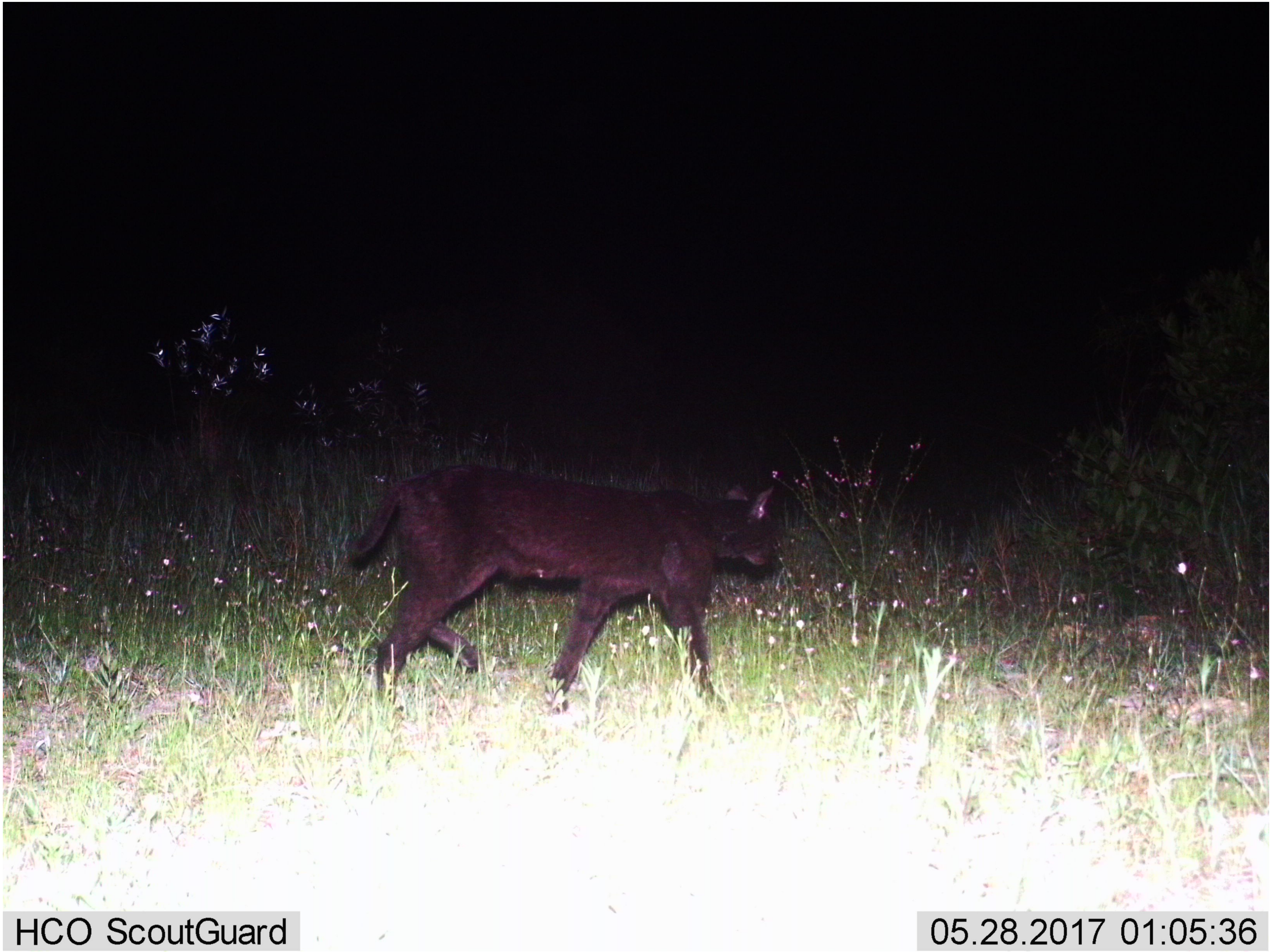
HCO ScoutGuard
05.28.2017 01:05:36

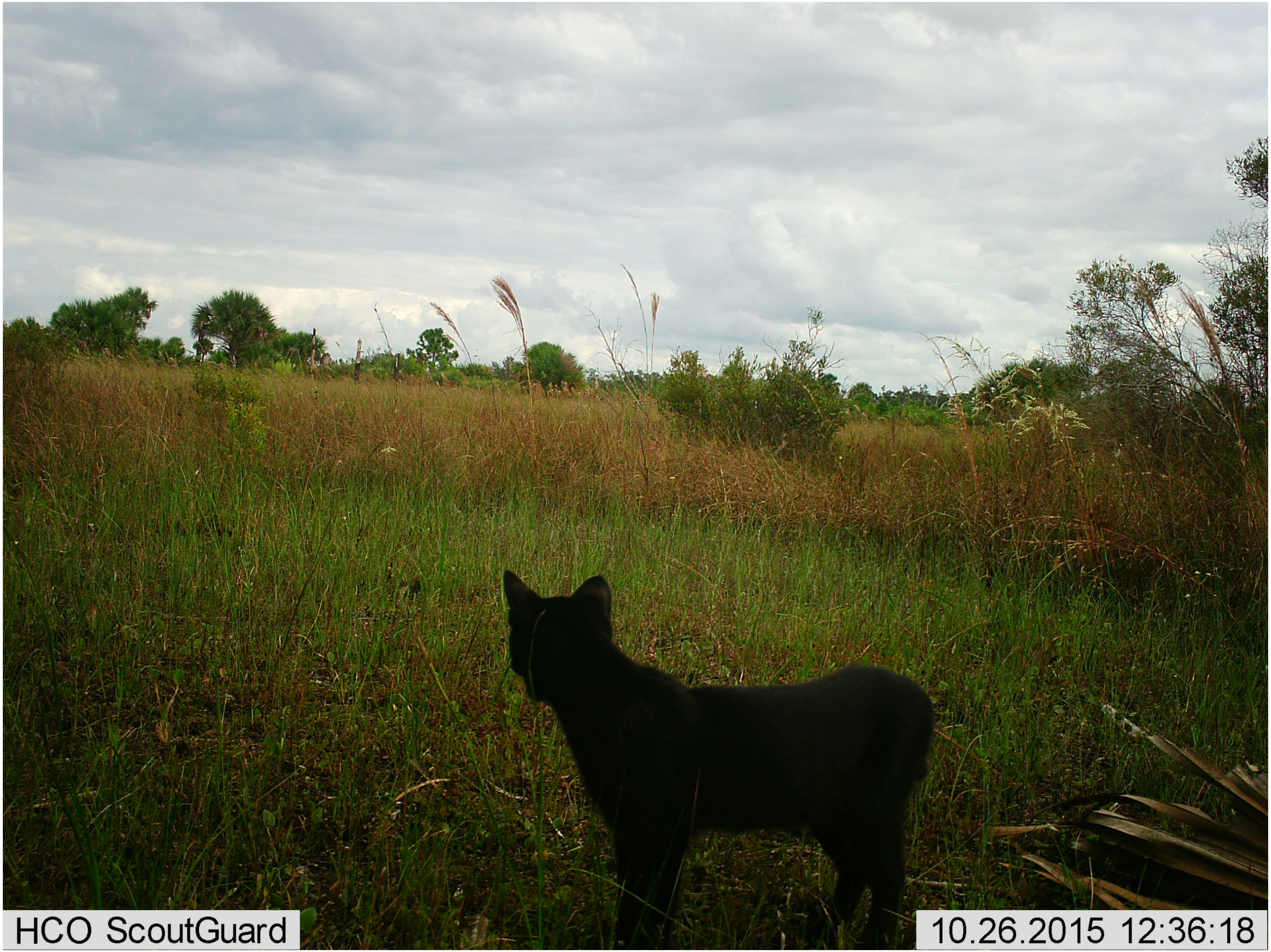
HCO ScoutGuard
10.26.2015 12:36:18

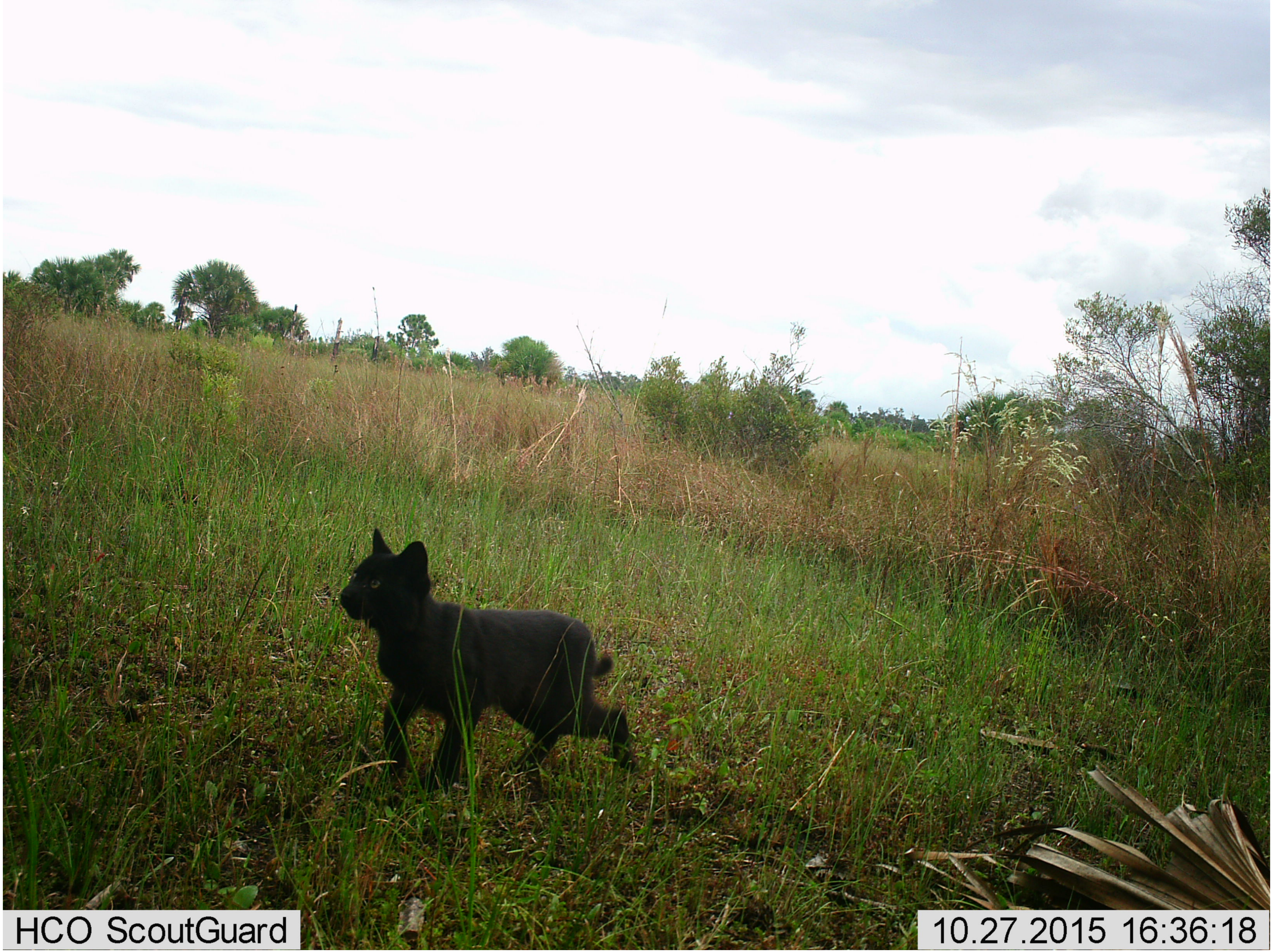
HCO ScoutGuard
10.27.2015 16:36:18
